# Multispectral sensing of biological liquids with hollow-core microstructured optical fibres

**DOI:** 10.1038/s41377-020-00410-8

**Published:** 2020-10-10

**Authors:** Timur Ermatov, Roman E. Noskov, Andrey A. Machnev, Ivan Gnusov, Vsevolod Аtkin, Ekaterina N. Lazareva, Sergei V. German, Sergey S. Kosolobov, Timofei S. Zatsepin, Olga V. Sergeeva, Julia S. Skibina, Pavel Ginzburg, Valery V. Tuchin, Pavlos G. Lagoudakis, Dmitry A. Gorin

**Affiliations:** 1 Skolkovo Institute of Science and Technology, 3 Nobelya str., Moscow, 121205 Russia; 2 Department of Electrical Engineering, Tel Aviv University, Ramat Aviv, Tel Aviv, 69978 Israel; 3 Light-Matter Interaction Centre, Tel Aviv University, Ramat Aviv, Tel Aviv, 69978 Israel; 4 Saratov State University, 83 Astrakhanskaya str., Saratov, 410012 Russia; 5 Tomsk State University, 36 Lenin’s av., Tomsk, 634050 Russia; 6 M.V. Lomonosov Moscow State University, Leninskie Gory, 1-3, Moscow, 119992 Russia; 7 SPE LLC Nanostructured Glass Technology, 101 50 Let Oktjabrja, Saratov, 410033 Russia; 8 Center for Photonics and 2D Materials, Moscow Institute of Physics and Technology, Dolgoprudny, 141700 Russia; 9 Institute of Precision Mechanics and Control of the Russian Academy of Sciences, 24 Rabochaya str., Saratov, 410028 Russia

**Keywords:** Optical sensors, Fibre optics and optical communications

## Abstract

The state of the art in optical biosensing is focused on reaching high sensitivity at a single wavelength by using any type of optical resonance. This common strategy, however, disregards the promising possibility of simultaneous measurements of a bioanalyte’s refractive index over a broadband spectral domain. Here, we address this issue by introducing the approach of in-fibre multispectral optical sensing (IMOS). The operating principle relies on detecting changes in the transmission of a hollow-core microstructured optical fibre when a bioanalyte is streamed through it via liquid cells. IMOS offers a unique opportunity to measure the refractive index at 42 wavelengths, with a sensitivity up to ~3000 nm per refractive index unit (RIU) and a figure of merit reaching 99 RIU^−1^ in the visible and near-infra-red spectral ranges. We apply this technique to determine the concentration and refractive index dispersion for bovine serum albumin and show that the accuracy meets clinical needs.

## Optical biosensing: analyzing biological fluids at many wavelengths

In-fibre multispectral optical sensing (IMOS) can detect and analyze the optical properties of biological liquids at multiple wavelengths simultaneously, using hollow-core microstructured optical fibres (HC-MOFs). Researchers in Russia and Israel, led by Dmitry Gorin at the Skolkovo Institute of Science and Technology in Moscow and Roman Noskov at Tel Aviv University, developed IMOS and demonstrated its potential using solutions of the bovin serum albumin. IMOS detects changes in the transmission of light in the fibre when the fluid being analyzed flows through the fibre’s hollow core. Refractive index values were measured at 42 wavelength’s of visible and near-infra-red light simultaneously, with potential to measure many more. IMOS is sufficiently accurate for clinical analysis, including detecting the presence and concentration of specific biomolecules in blood. The procedure could supply immediate diagnostic results relevant to a variety of medical conditions, including diabetes and cancer.

## Introduction

The increasing medical need for robust techniques suitable for real-time diagnostics at the place of patient care is at the cutting edge of modern biosensing^1^. Among the variety of available sensing devices, optical label-free sensors demonstrate high sensitivity to ambient refractive index (RI) variations, attracting considerable attention from the chemical, biomedical and food processing industries^2^. The main efforts in the development of such sensors are focused on increasing RI sensitivity (RIS) through the employment of cavity resonances (including surface plasmon resonances, Mie resonances, whispering gallery modes, etc.) and propagating eigenmodes in dielectric and plasmonic nanostructures^3^. Conceptually, another approach is realized by hollow-core microstructured optical fibres (HC-MOFs), which allow sensing of liquid analytes by monitoring the changes in the transmission; HC-MOFs have a great advantage of enabling high volume for measuring light-analyte interactions, which improves the RIS in comparison to that with cavity-based counterparts^4^.

Almost all optical sensors typically exploit a single resonance feature in the reflection/transmission/scattering spectra, following resonant shifts, associated with variations in the analyte RI and concentration^5^. Along with the specificity to target biomolecules supplied by functionalization of sensing template nanostructures with antibodies, aptamers and other analyte binders^6^, such biosensors may show very high sensitivity and figures of merit^7,8^. However, this strategy is time-consuming and quite expensive, and it disregards RI optical dispersion, which can act as a simple and cost-effective fingerprint of liquid biosamples to enable real-time monitoring of changes in their composition. In particular, variations in the optical dispersion of blood serum may indicate some diseases since it is directly related to changes in blood components, specifically the concentration of albumin and the appearance of its conjugated forms^9–12^. Recently, it was shown that variations in the blood serum RI could be used as an additional criterion in the analysis of antitumour therapy^13,14^, and RI monitoring for glycated haemoglobin and albumin enables diagnostics of type 2 diabetes and pre-diabetic status^15,16^.

Serum albumin is the most abundant blood plasma protein and plays a pivotal role in maintaining oncotic pressure as well as in transporting poorly soluble molecules, including lipid-soluble hormones, bile salts, unconjugated bilirubin and many others^17^. The normal concentration of albumin in the serum of human adults is 35–54 gL^−1^, while its deviations indicate various abnormal conditions and diseases^12^. Since albumin is optically transparent, direct determination of the analyte concentration by measuring the optical absorption is not applicable. The typical detection of the albumin concentration is based on the changes in dye absorbance (such as bromocresol green or bromocresol purple) upon binding to albumin^18^. Alternatively, it was proposed to detect low concentrations of albumin with the optical spring effect in an optomechanical oscillator^19^ and plasmon polaritons in a hyperbolic metamaterial^7^. Such techniques, however, are time-consuming and do not allow instantaneous monitoring of albumin in biological fluids in real time.

**Fig. 1:**
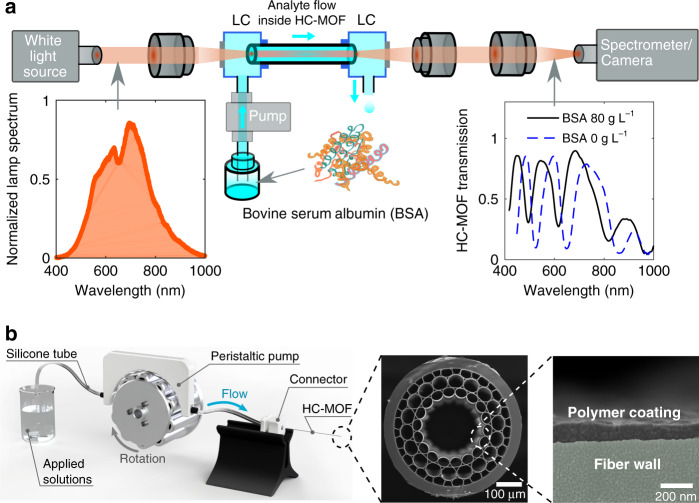
Illustration of the dynamic multispectral sensing concept for liquid samples. **a** Scheme of the setup for transmission characterization of HC-MOFs. Fibre facets are fastened in liquid cells (LCs), which are optically accessible via thin glass windows, allowing simultaneous pumping of fluids through fibre capillaries and measurement of the transmission spectrum. The red rays illustrate the light path from a broadband halogen lamp through the HC-MOF to the spectrometer and the CCD (charge-coupled device) camera to record the output mode profile. The insets depict the input and output spectra for different concentrations of BSA dissolved in PBS. **b** The setup for functionalization of HC-MOFs with LbL assembly. A peristaltic pump drives the flow of applied solutions (polyelectrolyte water solution and pure water) through the full fibre length, leading to the formation of the polymer coating on the inner surface of the core capillary. The procedure is repeated with inversely charged polyelectrolytes to create the desired number of bilayers. The insets depict the scanning electron microscopy (SEM) images of the fibre end face and the magnified capillary wall with the polymer coating formed by 18 PE bilayers

Here, we introduce the concept of in-fibre multispectral optical sensing (IMOS) for liquid biological samples in both static and real-time modes. The sensing principle relies on detecting spectral shifts of maxima and minima in the transmission spectrum of a hollow-core microstructured optical fibre when a liquid bioanalyte is streamed through it via specially designed liquid chambers (Fig. 1a). These resonant features are associated with Fabry–Perot resonances in the core capillary wall, and their spectral positions are unambiguously related to the bioanalyte RIs. A single fibre enables measurement of the RI at ~10 wavelengths with a sensitivity up to ~3000 nmRIU^−1^ and a figure of merit (FOM) reaching 99 RIU^−1^ in the visible and near-infra-red spectral domains. To increase the number of acquisition wavelengths to 42, we produce several HC-MOFs with slightly shifted transmission windows by coating their capillaries with polymer nanofilms of various thicknesses using the highly controllable and reproducible layer-by-layer (LbL) assembly of oppositely charged polyelectrolytes (PEs) (Fig. 1b). We demonstrate the practical performance of IMOS by measuring the concentration of bovine serum albumin (BSA) dissolved in water and in a phosphate-buffered saline (PBS) solution in both static and dynamic modes and show a resolution ~1 g L^−1^ when determining the BSA concentration, which matches the accuracy of standard tests on albumin^18^. Furthermore, for the first time, to our knowledge, we have extracted RI dispersion of pure BSA in a wide wavelength range of 400–850 nm. The important advantage of IMOS in comparison with many other optical biosensors is its simplicity and cost efficiency since it does not require any external cavity or interferometer, and the production of functionalized HC-MOFs is simple and inexpensive. In addition, IMOS makes it possible to perform RI measurements within a wide spectral domain in real time, which is still a challenging issue for other alternative methods.

## Results

### Principle of in-fibre multispectral optical sensing (IMOS)

To measure the optical transmission of liquid-filled HC-MOFs, we employ the setup shown in Fig. 1a. Liquid filling of fibre capillaries is obtained by inserting the tips of HC-MOFs into small liquid cells (LCs) equipped with tubing interfaces and optically transparent windows^4^. The LCs are 3D-printed from acrylonitrile butadiene styrene and exhibit a dead volume of ~50 μL (see the Supplementary information). This value is sufficiently small for stable and reproducible measurements in both static and dynamic regimes (at least at the pumping rate of ~1 mL min^−1^ or slower). The flow of the analyte through the HC-MOF is driven by a peristaltic pump (Shenchen LabV1) connected to the inlet of the LC. Microscope objectives (Olympus, 10×) are used to couple the light from a halogen lamp (Thorlabs SLS201L) into the fibre and to collect the transmitted signal. The output signal is analyzed with a spectrometer (Ocean Optics QE Pro) combined with a CCD camera (Thorlabs DCU223C) (see further details in “Materials and methods”).

The employed soft-glass HC-MOFs have a considerably thick wall of the central capillary of 1.82 μm (see Fig. S1) so that the light guiding mechanism can be described via Fabry–Perot resonances^20,21^. In accord with this model, the resonant coupling between the main core (guiding) mode and the capillary (cladding) modes corresponds to the maxima (Eq. (1)) and minima (Eq. (2)) in the fibre transmission, which occur at

1 $$\lambda _{j\,{\rm{max}}} = \frac{{4d}}{{2j + 1}}\sqrt {n_2^2 - n_1^2}$$

2 $$\lambda _{j\,{\rm{min}}} = \frac{{2d}}{j}\sqrt {n_2^2 - n_1^2}$$

where *j* is an integer describing the capillary mode order (*j* = 1, 2, 3, …), *n*_1_ is the RI of an analyte filling the capillaries, *n*_2_ is the RI of the fibre glass and *d* indicates the wall thickness for the first capillary layer. This relation provides a convenient link between the RIs of an analyte and the maxima and minima in the fibre transmission. Specifically, for *d* = 1.82 μm, the HC-MOF filled with water exhibits four transmission windows in the wavelength range 400–900 nm, and the minima appearing at 411, 494, 617 and 821 nm for *j* = 6, 5, 4 3 obey Eq. (1). However, since the transmission windows can be asymmetric due to non-negligible dispersion of the fibre glass and other optical elements (Fig. 1a), the positions of the centroids have been found to be more sensitive to the variations in an analyte RI than the maxima, and we use them for further measurements.

Thus, a single fibre allows simultaneous measurement of an analyte’s RI synchronously at seven wavelengths in the visible and near-infra-red spectral domains. However, such a number of wavelengths is still insufficient for an accurate retrieval of RI optical dispersion, especially in cases when data are contradictory or not available. The number of discrete wavelengths available for measuring RI can be increased by using several HC-MOFs with slightly shifted transmission windows. However, reproducibly drawing such a family of fibres with variations in *d* ~ 10 nm is technically quite a challenging task that would inevitably increase the cost of IMOS. In the next section, we resolve this issue by presenting a simple and inexpensive technique that allows a small increase in the thickness of capillary walls in a reproducible and highly controllable way.

### Functionalization of hollow-core microstructured optical fibres with LbL assembly

The basic principle of our approach is the LbL assembly of oppositely charged PEs onto the glass surface. Figure 1b shows the setup used for coating the fibre capillaries^22^. The peristaltic pump creates a flow of PE solutions through the fibre with a controllable and persistent rate for a pre-determined volume. This procedure results in the uniform deposition of PE layers inside the fibre capillaries. As PEs, aqueous solutions of polycationic poly(allylamine hydrochloride) (PAH) and polyanionic poly(styrenesulfonate) (PSS)^23^ have been used.

The coating procedure can be described as follows. First, the HC-MOF is rinsed with deionized water for 2 min at a flow rate of 500 μL min^−1^ to remove dust particles prior to PE deposition. The very first deposited PE layer consists of polyethylenimine (PEI) serving as an adhesive or anchor agent that provides a high surface charge density with a homogenous distribution due to its high molecular weight and branched structure^24,25^. For each layer, we coat HC-MOF sequentially by PAH and PSS PE solutions (each at a concentration of 2 mg mL^−1^) for 7 min. To ensure successful adsorption and to prevent colloid depletion during the multistep LbL deposition process, we use concentrated PE solutions (2 mg mL^−1^) that allow exceeding the minimum threshold for molecule attachment and reversing the charge polarity for each adsorbed layer^26,27^. Finally, we remove unbound polymer molecules and prevent cross-contamination of the solutions by washing the samples with pure deionized water after each layer is deposited^28^.

### Ionic strength influence on the polymer coating thickness

It has been demonstrated that the thickness of LbL-assembled layers on a planar substrate is proportional to the square root of the ionic strength^29^ that, in turn, is the square function of the molar concentration of ions. Hence, the ionic strength is typically supported by sodium and chlorine ions, which are the main components of blood plasma as well as saline solution. Neff et al.^29^ demonstrated that an increase in the buffer molar concentration yields thicker LbL-assembled PAH/PSS multilayers. For example, the thickness per single layer is 1.3 ± 0.1 nm at 0.05 M NaCl but is 2.2 ± 0.1 nm at 0.5 M NaCl^29^. In addition, the higher concentration of sodium chloride gives rise to an increased roughness caused by altering the molecular conformation from a linear to globular structure^30^.

**Fig. 2:**
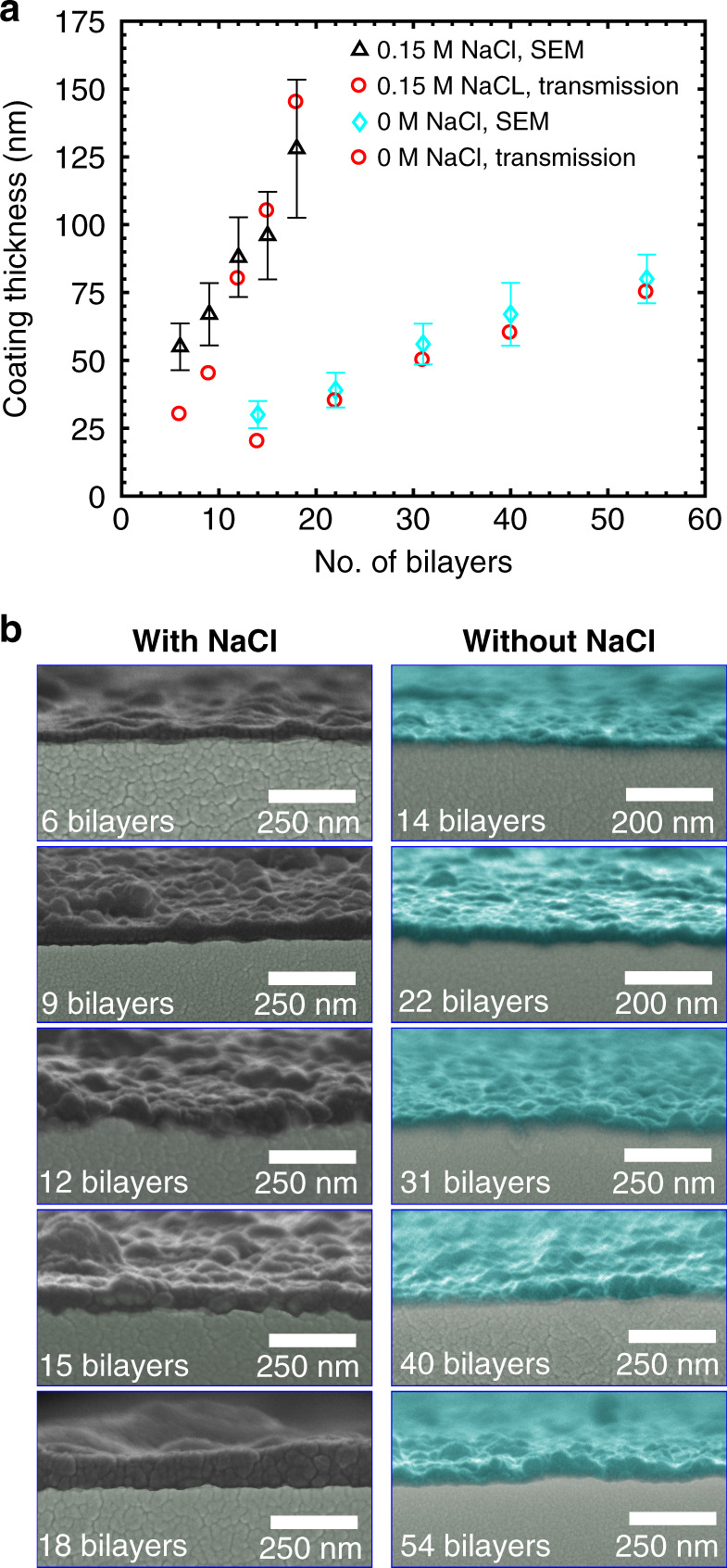
Comparison of the capillary polymer coatings obtained by the PEs dissolved in deionized water and in buffer containing 0.15 M NaCl. **a** Polymer coating thickness versus the number of PE bilayers. The coating thickness is evaluated from SEM images (SEM) and obtained by fitting the shifts in the fibre transmission spectra through the condition of Fabry–Perot resonances (transmission) (see the Supplementary information). Polymer coatings are included in the theoretical model as additional concentric layers of equal thickness on the core capillary. The thickness is fitted to obtain the coincidence of the minima positions for both the experimental and calculated transmission spectra. Error bars show the coating roughness. **b** SEM micrographs of the core capillaries functionalized with different numbers of PE bilayers. Left column: PEs in deionized water with 0.15 M NaCl. Right column: PEs in pure deionized water. Pseudocolour indicates the polymer coating

To gain insight into the impact of the solution ionic strength on the coating performance, we systematically compare the thickness and morphology of assembled PAH/PSS films with PEs at the same concentration dissolved in deionized water and in buffer containing 0.15 M NaCl. Figure 2a shows the thickness of the coatings prepared by the alternating deposition of PAH and PSS layers from PE solutions with and without NaCl as a function of the number of deposited PE bilayers. The coating thickness linearly depends on the number of assembled PAH/PSS bilayers. However, the average thickness increase is 1.8 ± 0.3 nm and 7.0 ± 1.3 nm per bilayer for the salt-free PE solutions and in the presence of 0.15 M NaCl, respectively. These values differ from previously published data for LbL coating of planar substrates, where the average thickness per PE layer was ~1–3 nm^23,31–33^. We attribute this discrepancy to different hydrodynamic conditions for PE adsorption in our study^22^ and the effect of the charged capillary surface. We also observe an increase in the film thicknesses with increasing ionic strength in the PE solutions. This result is in good agreement with previously published works on LbL coating of planar substrates^23,31,32^.

In addition, we evaluate the morphology of PE layers by SEM micrographs of fibre cross-sections (Fig. 2b). For a moderate number of bilayers, salt-free PE solutions result in a relatively thin and smooth structure of the coating because of the self-adjustment of highly flexible polymeric chains^23^ as well as the linear molecular conformation. With an increasing number of deposited PAH/PSS bilayers, quasi-spherical structures appear on the coating surface as a result of PE aggregation and lead to increased roughness, which, however, does not exceed 25 nm for the coating formed by 54 polymer bilayers. In contrast, the presence of NaCl in the PE solutions markedly modifies the structure of the polymeric molecules by a conformational transition from extended polymeric chains to globular structures, leading to the formation of polymer bundles with sizes up to hundreds of nanometres (Fig. 2b). Hence, PEs adsorbed from the saline buffer create a rigid quasi-spherical structure of coating with an average roughness of 50 nm for the coating formed by 18 PAH/PSS bilayers, giving rise to extra scattering for guiding light and enhancing the fibre optical losses. However, this coating does not significantly affect the transmission performance of functionalized HC-MOFs (Fig. S4 in the Supplementary information) since this roughness is much smaller than the light wavelength.

These results demonstrate that the structure of LbL-assembled PAH/PSS films is highly dependent of the salt concentration in the PE solutions, and the coating thickness can be tailored in a reproducible and controllable way by varying the number of PE bilayers deposited. The salt-free PE solution provides the smallest roughness and finer tuning of the transmission windows, so we use HC-MOFs functionalized with up to 54 bilayers by this protocol for IMOS.

It is instructive to note that LbL-assembled PAH/PSS films in HC-MOFs show extremely robust stability under various conditions, including long-term storage (Fig. S8), a wide temperature range from 22 to 120 °C (Fig. S9), pH levels from 4 to 10 (Fig. S10) and various ionic strengths (Fig. S11) of the sample liquid along with subsequent washing of functionalized HC-MOFs by water and drying.

### Optical transmission of functionalized HC-MOFs

**Fig. 3:**
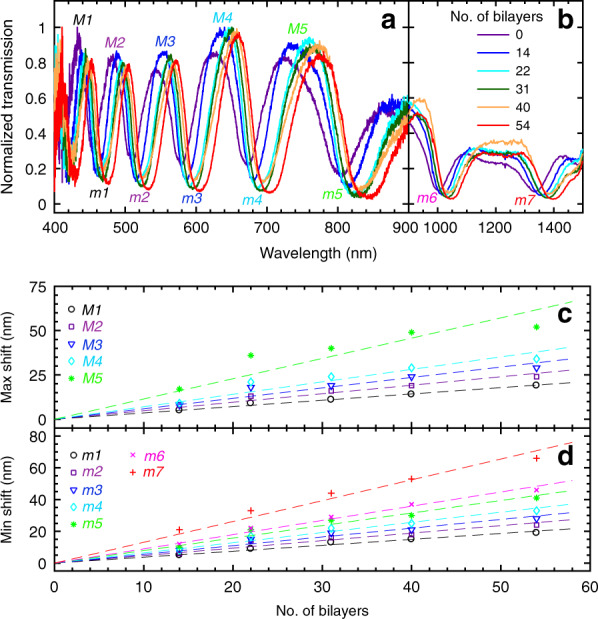
Optical characterization of HC-MOFs functionalized by PEs adsorbed from the salt-free buffer. **a**, **b** Transmission spectra in the visible (400–900 nm) and near-infra-red (900–1500 nm) ranges. The results for 6 samples with various numbers of PE bilayers are presented. Note the different scales along the *x* axis for (**a**) and (**b**). Shifts of maxima (**c**) and minima (**d**) of the transmission along with the linear fits

HC-MOF functionalization leads to fine tuning of their optical transmission windows, as shown in Fig. 3. Specifically, shifts in maxima and minima of the transmission appear to be almost a linear function of the number of bilayers. This fact results from the similar RIs of the fibre glass^34,35^ and PAH/PSS^36,37^; thus, polymer coatings can be considered an effective instrument to vary the thickness of the core fibre capillary. Hence, the spectral positions of the transmission maxima and minima can be described by Eqs. (1) and (2), which, assuming a 1.8-nm thickness for every bilayer, yield the linear fits shown in Fig. 3c, d.

**Fig. 4:**
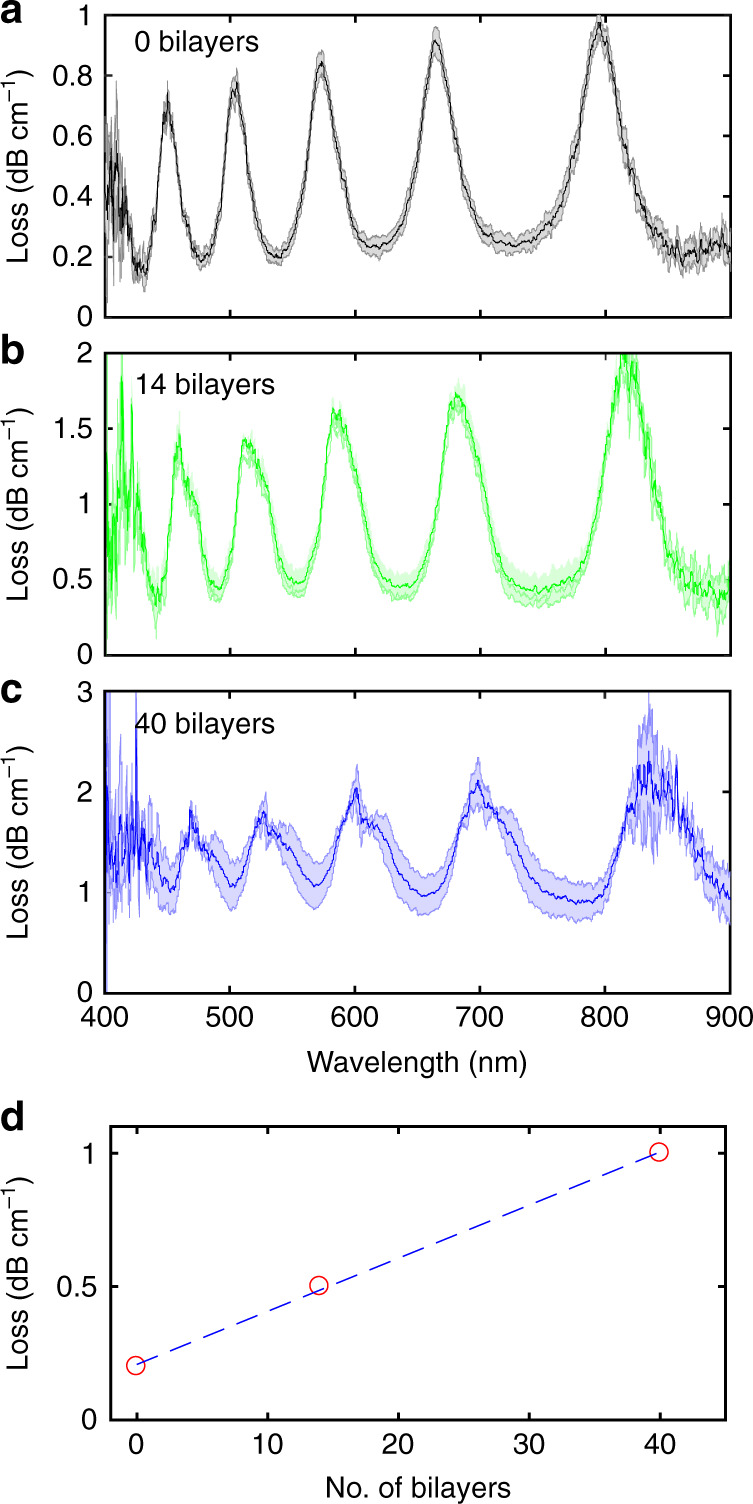
Characterization of optical losses in functionalized HC-MOFs by the cut-back technique. **a** Unmodified MOF. **b**, **c** Fibres coated with 14 and 40 PE bilayers in the salt-free solution. Shadow areas show the error range. Loss peaks fit Eq. (2). **d** The minimal optical loss in the transmission windows as a function of the bilayer quantity

Importantly, LbL functionalization results in additional fibre losses. The results of cut-back measurements for the samples modified in the salt-free PE solution are presented in Fig. 4 (see also “Materials and methods”). One can observe that the polymer coating leads to an average extra attenuation of ~0.02 dB cm^−1^ per single assembled PAH/PSS bilayer. However, this issue does not significantly disturb light guidance inside the fibre as long as the number of bilayers is limited.

### IMOS in static mode

**Fig. 5:**
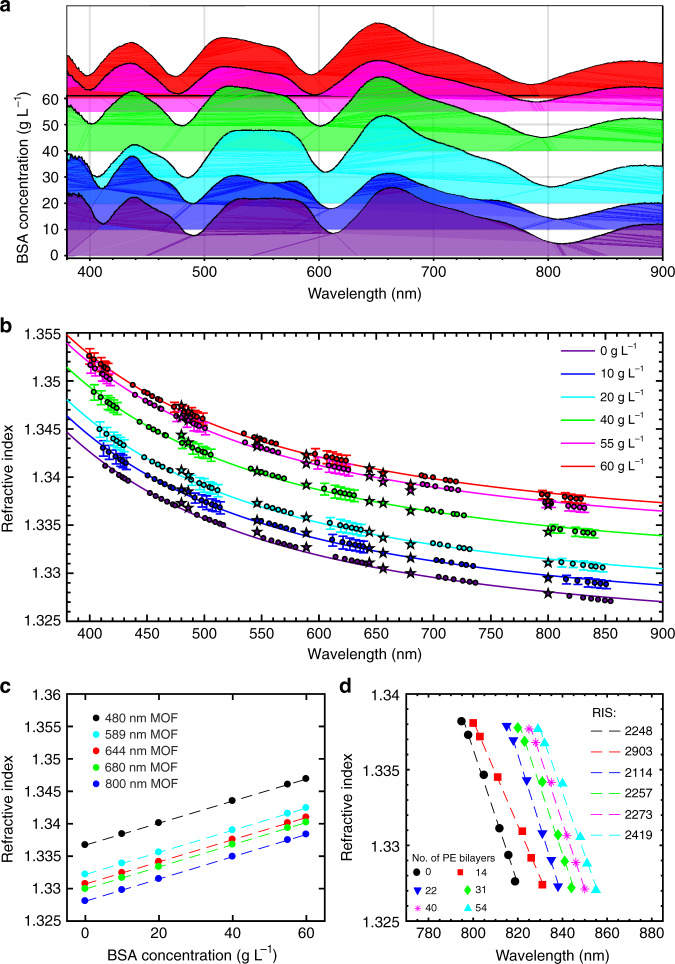
Performance of IMOS for BSA dissolved in deionized water in static mode. **a** Transmission spectrum evolution with increasing BSA concentration for the non-modified HC-MOF. **b** Optical dispersion of BSA in a water buffer with different concentrations. The coloured points indicate the RI values extracted from the shifts in the minima and the peak centroids of the fibre transmission spectra, and the solid lines mark their Sellmeier fits. For pure water, the Sellmeier fit describes the water dispersion adopted from ref. ^38^. The error bars are obtained from the spectrometer optical resolution. The stars mark RIs measured by the Abbe refractometer, provided for comparison. **c** Calibration relating the concentration of BSA and RIs of the BSA-water solution at different wavelengths. The RIs of BSA are extracted from the Sellmeier fits of the experimental points (see Table 1S). **d** The positions of the longest-wavelength minima as functions of RI extracted from (**a**) and (**b**). The error bars are smaller than the data points

We demonstrate IMOS by measurements of the RI for BSA dissolved in a water buffer with the help of six functionalized HС-MOFs with 0, 14, 22, 31, 40 and 54 PE bilayers. To simplify the interchange of fibres during the measurement, we replace the LCs in the setup (Fig. 1a) with a custom smart cuvette^21^ (“Material and methods”). We start with calibration of the system by recording the transmission of the water-filled fibre (Fig. 5a) and associate the spectral positions of the minima and the peak centroids with the water RIs by using Eqs. (1) and (2). Each fibre provides RIs at seven discrete wavelengths (four minima and three peak centroids) that are increased to 42 points due to the employment of six functionalized fibres with slightly shifted transmission windows. Thus, the validity of our approach is confirmed by the excellent agreement of our measurements with the well-known optical dispersion of deionized water adopted from ref. ^38^ (Fig. 5b).

Figure 5a shows the evolution of transmission spectra caused by an increase in BSA concentration in a water buffer. Using these data, we extract RIs by the same technique as that for pure water filling and plot them along with the Sellmeier fits, as shown in Fig. 5b. To verify our results independently, we also measure the RIs of these samples by a multiwavelength Abbe refractometer (denoted by the stars in Fig. 5b) and find very good agreement with the IMOS results.

In practical applications, it is important not only to determine RIs of a biological liquid but also to quantify the concentration of target biomolecules. To this end, we calibrate our system by associating the RIs of the samples at different wavelengths with the concentration of BSA (Fig. 5c and Table S3). Since the concentration of BSA is quite low, these dependencies are linear.

However, it should be noted that solutions with strongly absorbing analytes can be investigated only with preliminary preparation of the probe via dilution. The higher optical losses introduced by the low-transparency filling media result in minima and maxima distortion (see the Supplementary information).

### RIS and FOM

The key characteristics of label-free optical sensors are the RIS, defined as the ratio of the change in sensor output (the shift of the resonant wavelength) to the analyte RI variation, and the FOM, which normalizes the RIS to the width of the tracked resonance characterized by the full width at half maximum^2^. Using the fibre transmission spectra (Fig. 5a) and the determined RIs of the BSA-water solutions (Fig. 5b), we plot the transmission spectra minima versus the analyte RI (see Fig. 5d for the longest-wavelength minima and Fig. S15 for all minima). The slopes of the linear fits show the RIS of our sensor, which varies from 1100 nm RIU^−1^ for blue light to 3000 nm RIU^−1^ for infra-red light. The corresponding FOM varies from 60 to 99 RIU^−1^. These values along with the working spectral range are typical for surface plasmon and 2D material sensors^2^. However, our approach provides a great benefit of multispectral analysis in a broad spectral range.

### Optical dispersion of BSA

**Fig. 6:**
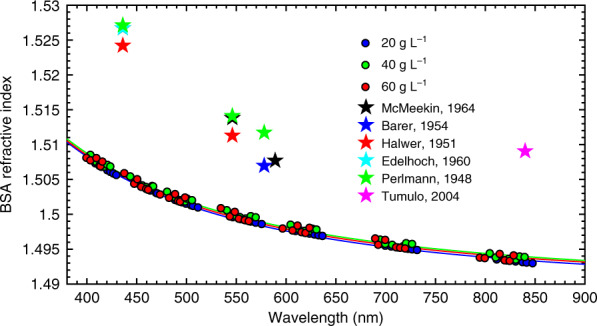
The refractive index of BSA determined via the Maxwell Garnett approximation. Points correspond to the measurements of RI for BSA-water, and solid lines mark the Sellmeier fits (see the Supplementary Information, Table S4). The extraction was performed for three concentrations of BSA to show the repeatability of the result. The error bars are smaller than the marker size. The stars mark BSA RI values adopted from refs. ^39–43,45^

It is important to note that the optical dispersion for a solution containing an investigated substance dissolved in a water buffer can be used to determine the optical dispersion of the pure product. Such a possibility is of special interest in cases when the substance of interest is difficult to synthesize in the form of thin films, which are normally suitable for analysis with standard techniques such as ellipsometry or refractometry. This is the case for BSA, whose RI has been reported for only a few wavelengths: 436^39–42^, 546^39,40,42^, 578^42–44^, 589^39,44^ and 840 nm^45^. Accounting for BSA molecules as uniaxial ellipsoids with overall dimensions of 4 nm × 4 nm × 14 nm^46,47^, the extraction can be performed by using the effective medium approximation, which describes the effective permittivity *ε*_eff_ of the solution by the Maxwell Garnett equation as follows^48,49^:

$$\varepsilon _{\rm{eff}} = \varepsilon _{\rm{e}} + \varepsilon _{\rm{e}}\frac{{\frac{{f_{\rm{BSA}}}}{3}\mathop {\sum}\nolimits_{j = x,y,z} {\frac{{\varepsilon _{\rm{i}} - \varepsilon _{\rm{e}}}}{{\varepsilon _{\rm{e}} + N_j(\varepsilon _{\rm{i}} - \varepsilon _{\rm{e}})}}} }}{{1 - \frac{{f_{\rm{BSA}}}}{3}\mathop {\sum}\nolimits_{j = x,y,z} {\frac{{N_j(\varepsilon _{\rm{i}} - \varepsilon _{\rm{e}})}}{{\varepsilon _{\rm{e}} + N_j(\varepsilon _{\rm{i}} - \varepsilon _{\rm{e}})}}} }}$$

where *ε*_*e*_ and *ε*_*i*_ are the permittivities of the water buffer and the inclusions (BSA molecules), respectively, *f*_BSA_ is the volume fraction of the BSA molecules, and *N*_*x*_*, N*_*y*_ and *N*_*z*_ are the depolarization factors over the *x*, *y* and *z* axes, respectively. The molecular weight of 66.5 kDa^46,47^ and dimensions of BSA molecules allow us to translate the mass concentration of BSA into the volume filling factor *f*_BSA_. By using the known optical dispersion of water and the measured RIs of the BSA-water mixtures, we determine the optical dispersion of BSA (Fig. 6). To verify the repeatability of this process, we perform this procedure for three concentrations of BSA, 20 g L^−1^ (*f*_BSA_ = 0.0213), 40 g L^−1^ (*f*_BSA_ = 0.0425), and 60 g L^−1^ (*f*_BSA_ = 0.0638), resulting in almost identical dispersion curves. The discrepancy falls within the range of the measurement accuracy.

It is instructive to compare the RI dispersion of BSA obtained by IMOS with the data available in the literature^39–43,45^ (Fig. 6). All previous measurements of BSA RI have been performed by refractometry of water-BSA solutions, and they show deviations from each other and from our results. However, the difference between the results is in the range of less than 0.025 RIU. These dissimilarities can be attributed to the distinct purity of BSA used in different measurements. In our experiments, we utilize BSA produced by Sigma-Aldrich with a purity above 96%, while the purity of BSA measured in previous works was not reported. However, the purity is a critical parameter that can significantly affect the measurement results. To this end, we compare the results for BSA produced by Sigma-Aldrich (>96% purity)^50^ (St. Louis, MO, USA) and Agat-Med (50% purity)^51^ (Moscow, Russia) and find a discrepancy of ~0.05 RIU (Fig. S16). The purity of the samples has been verified by sodium dodecyl sulfate-polyacrylamide gel electrophoresis analysis (Figs. S17 and S18).

### IMOS in real time

**Fig. 7:**
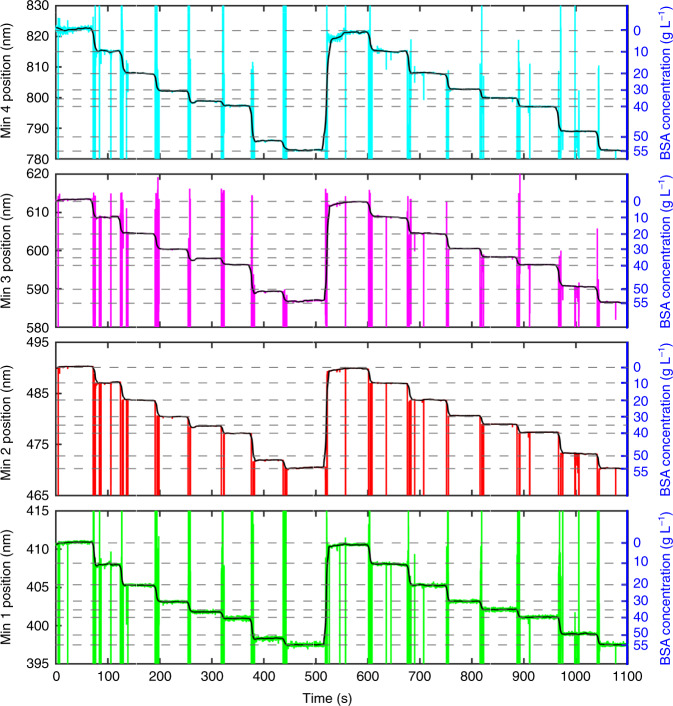
IMOS in real time for BSA dissolved in PBS buffer. The coloured lines denote instantaneous positions of the transmission minima when the peristaltic pump is switched between five solutions with different concentrations of BSA. The black lines exhibit the data smoothed with MATLAB. The flow rate created by the peristaltic pump is fixed at 1 mL min^−1^

To demonstrate IMOS in a dynamic regime, we return to the setup where the HC-MOF is sealed in LCs (Fig. 1a). The experiment is organized as follows: we subsequently switch the peristaltic pump between seven solutions with different concentrations of BSA in a PBS buffer and use the specially developed algorithm in LabVIEW to track in real time the spectral positions of minima in the fibre transmission spectra. Figure 7 displays the results. Although the PBS buffer for BSA dissolution prevents the intensive formation of air bubbles while the analyte flows through the fibre, bubbles still appear at the moments of solution switching, leading to instantaneous disturbance of the minima positions during the transitional time intervals (coloured curves in Fig. 7). To circumvent this effect, we process the data by smoothing and present the results as black curves in Fig. 7. One can observe instantaneous shifts in the minima positions on the spot in response to variations in the analyte concentration. In turn, the analyte RIs can be determined via calibration in Fig. 5c. The repeatability of the measurements is evidenced by the two equal cycles of transitions.

In addition to exhibiting high repeatability, the dynamic test shows the real-time RI measurements in a wide spectral domain, and the response speed of the proposed sensor can be treated as instantaneously fast. However, considering the specific measurements, we should take into account the time needed for target molecules or particles to bind onto the inner surface of the fibre core. Thus, IMOS in real time is a powerful technique enabling instantaneous determination of the analyte optical dispersion and concentration. Fibre sealing into LCs connected to the peristaltic pump enables further integration into various optofluidic systems that demand fast, precise and easy sensing tools (Fig. 1). Considering their reusability and high stability to various environmental conditions, functionalized HC-MOFs can be used for the analysis of various biological liquids, and the cleaning of HC-MOFs can be ensured by the water flow, which removes the remaining solutions and adsorbed molecules.

It should be noted that real-time monitoring of RI optical dispersion simultaneously at 42 (or even several hundred) wavelengths can be achieved by using several post-processed HC-MOFs with necessary modifications of LCs and adaptation of the optical setup to parallel measurements (Figs. S5 and S6).

## Discussion

In summary, we have proposed and experimentally demonstrated the technique of IMOS based on the analysis of spectral shifts in the minima and maxima of the HC-MOF transmission filled with a bioanalyte. In contrast to conventual optical biosensors that work at only a single wavelength, our approach allows simultaneous measurement of the RI at many wavelengths in the visible and near-infra-red spectral domains with RIS values up to ~3000 nm RIU^−1^ and FOMs reaching 99 RIU^−1^. Specifically, we have demonstrated the use of IMOS to measure the optical dispersion and concentration of BSA dissolved in water and PBS buffers in both static and dynamic regimes, with a resolution of ~1 g L^−1^ when determining the BSA concentration, which matches the accuracy of the standard tests on albumin^18^. Furthermore, for the first time, to our knowledge, we have extracted the RI of pure BSA at 42 spectral points in the wavelength range 400–850 nm. Potentially, the number of wavelengths available for measurement of RI can be increased further to several hundred by producing the necessary number of HC-MOFs with slightly shifted transmission windows. To this end, we have adapted the LbL technique to accurately coat fibre capillaries with polymer nanofilms. We have considered the effect of salt presence in the applied PE solutions on the coating performance and found that compared with the salt-enriched PEs, the salt-free PE solutions yield the smallest roughness and coating thickness per single bilayer. The proposed LbL deposition technique can be extended further, allowing one to reach novel sensing capabilities including HC-MOF functionalization by specific molecules to capture biomolecules (DNA, antibodies, aptamers, Fab, etc.) and target particles^52–54^.

It is instructive to compare IMOS with refractometers and ellipsometers, which perform similar RI identification. The operational principle of conventional Abbe refractometers relies on using a set of filters, one filter per single acquisition wavelength, so that the RI measurements can be performed in a static regime only, and the number of wavelengths available for the measurements is restricted by the number of filters. Modern digital refractometers are more convenient, performing all operations in an automatic mode; however, they do not support real-time measurements. In-line refractometers, which are widely used in various manufacturing areas, target only a single wavelength (usually 589 nm) and therefore cannot be used for the laboratory analysis of liquids over a wide spectral range. Overall, the modern market of available refractometers is diverse. However, these devices are expensive, restricted in the number of wavelengths available for measurement (typically less than 10) and cannot perform real-time measurements at several wavelengths in parallel.

The great advantage of ellipsometers is the ability to measure the RI in an ultrawide spectral band. However, ellipsometers are very expensive and bulky and require a complicated calibration process for every single measurement, so they cannot be easily integrated into any optofluidic or other sensing system to ensure real-time measurements.

Thus, the main benefits of IMOS in comparison to refractometers and ellipsometers are simplicity and cost efficiency, and the setup is quite compact and simple for reproduction. In addition, IMOS makes it possible to perform RI measurements over a wide spectral range, providing great capacity for practical use. Specifically, integration of LCs with surgery endoscopes will pave the way towards intraoperative analysis of bodily fluids, enabling surgeons to act in a timely manner under variable conditions. Multispectral analysis of saliva, urine, and ascitic fluid will facilitate diagnostics of various diseases. In addition, IMOS enables accurate determination of the RI optical dispersion for various proteins and their complexes, which is important for reliable simulations of biological processes.

## Materials and methods

### HC-MOF sample fabrication

We used HC-MOFs containing three concentric capillary layers surrounding a central hollow-core drawn from custom-made soft glass (Fig. 1b). The diameter of the central capillary is ~240 μm, the outer diameter is ~600 μm, the wall thickness of the first layer of capillaries is 1.82 μm, and the length of all samples is 6 cm. The geometrical features of the HC-MOF are detailed in Fig. S1. The spectral properties of these fibres have been discussed in refs. ^20,21^.

### Chemical reagents

All applied PEs and PBS were purchased from Sigma-Aldrich: PAH (MW = 50,000), PSS (MW = 70,000), and PEI (MW = 2,000,000). Deionized water was produced by a Millipore Milli-Q Plus 185 system. The BSA used in static dynamic measurements was supplied by Agat-Med and Sigma-Aldrich.

### Multilayered deposition process

HC-MOFs were connected to a peristaltic pump (Shenchen) by a flexible silicone tube with an inner diameter of 1 mm^22^. To fix fibre samples, we produced special 3D-printed clamps, which ensured the efficient flow of solutions through the fibres. Our system supplies a highly controllable and persistent flow rate for any given solution capacity, allowing the uniform deposition of PE layers inside the capillaries. Prior to PE layer assembly, the fibres were washed with deionized water for 2 min with a speed of 500 μL min^−1^ to ensure that small dust particles were removed; then, the fibres were subjected to the LBL technique by a combination of inversely charged PEs (PAH/PSS) with a concentration of 2 mg mL^−1^ in both cases: in the presence of 0.15 M NaCl in the PE solution and in pure deionized water. The pump flow rate was set to 150 μL min^−1^, and the amount of PE solution used for a single PE layer formation was 1 mL, which corresponded to an ~7 min deposition cycle. Deionized water was applied after each deposited PE layer with a speed of 200 μL min^−1^ for 2 min to wash the samples and to remove unadsorbed molecules. The very first bilayer of PEI/PSS was followed by the desired number of PAH/PSS bilayers to finalize the coating formation.

### Optical characterization of functionalized HC-MOFs

The output light of a broadband halogen lamp (Thorlabs SLS201L, 360–2600 nm) was initially collimated (Thorlabs F220SMA-532) and then focused by a 10× objective (Olympus) to the fibre input. The other 10× objective (Olympus) was used to collect the transmitted light, which was further guided to either a compact CCD spectrometer (Ocean Optics QE Pro) operating in the extended wavelength region (350–1000 nm) or an IR spectrometer (Ocean View NIRQuest). All of the transmission spectra were initially normalized to the spectrum of the halogen lamp and then to its maximum value. A colour CCD camera (Thorlabs DCU223C) in the collection part was installed to control the coupling conditions and the fibre end face cleaving quality and to record the output mode profile. The analysis of the losses induced by deposited PE layers was performed by the cut-back method. The light was coupled into the fibre, and four cut-back steps were performed with a 6-cm-long sample under fixed coupling. The purity of the fundamental mode was controlled by the CCD camera.

### Multispectral sensing of albumin solutions

In the static regime, HC-MOFs were integrated into a smart cuvette and filled by the solutions in the test through capillary action. Once the fibre was filled completely and no air bubbles appeared, the transmission spectrum was recorded successively for each fibre sample. In the dynamic regime, the fibre was sealed in specially designed LCs. A detailed schematic of the designed LCs is illustrated in Fig. S14. Liquid samples were streamed through the HC-MOFs by the peristaltic pump connected to the inlet of the LC. Real-time tracking of the transmission spectra minima was achieved by means of LabVIEW software. We used two spectrometers (Ocean Optics QE Pro) to cover the visible and IR spectral ranges.

### BSA refractometric measurements

The control measurement of RI for BSA (Fig. 6) was carried out by a multiwavelength Abbe refractometer (DR-M2/1550, Atago, Japan). The multiwavelength Abbe refractometer allows one to measure the RI in the wavelength range of 450–1550 nm with an accuracy of ±0.0002. As the source of radiation, we used a high-power incandescent lamp. The wavelength of light was determined by narrow band-pass filters with transmission windows of 480 ± 2 nm, 486 ± 2 nm, 546 ± 2 nm, 589 ± 2 nm, 644 ± 2 nm, 656 ± 2 nm, 680 ± 5 nm and 800 ± 5 nm. At the beginning of the experiment, the device was calibrated by measuring the RI of the prism (*n* = 1.3327) at a wavelength of 589 nm (the absorption band of sodium). Monobromonaphthalene was used as a contact liquid. The average measurement error of the RI was ±0.0003. The prism temperature during the measurements was kept at +24 °С by means of water circulation in the refractometer.

## Supplementary information

Supplementary information
