# Supplementary material for: Multispectral sensing of biological liquids with hollow-core microstructured optical fibres
Source: Light Sci Appl. 2020 Oct 10;9:173. doi: 10.1038/s41377-020-00410-8 (PMC7548008; doi:10.1038/s41377-020-00410-8)
Supplement: Supplementary file 1 — Supplementary information [file 41377_2020_410_MOESM1_ESM.docx]

**Supplementary information**

**Multispectral sensing of biological liquids** **with hollow-core microstructured optical fibres**

Timur Ermatov^1,^^, Roman E. Noskov^2,3,^^, Andrey A. Machnev^2,3^, Ivan Gnusov^1^, Vsevolod Аtkin^4^, Ekaterina N. Lazareva^4,5^, Sergei V. German^1^, Sergey S. Kosolobov^1^, Timofei S. Zatsepin^1,6^, Olga V. Sergeeva^1^, Julia S. Skibina^7^, Pavel Ginzburg^2,3,8^, Valery V. Tuchin^4,5,9^, Pavlos G. Lagoudakis^1^, Dmitry A. Gorin ^1,*^

^1^ Skolkovo Institute of Science and Technology, 3 Nobelya str., Moscow 121205, Russia

^2^ Department of Electrical Engineering, Tel Aviv University, Ramat Aviv, Tel Aviv 69978, Israel

^3^ Light-Matter Interaction Centre, Tel Aviv University, Ramat Aviv, Tel Aviv 69978, Israel

^4^ Saratov State University, 83 Astrakhanskaya str., Saratov 410012, Russia

^5^ Tomsk State University, 36 Lenin’s av., Tomsk 634050, Russia

^6^ M.V.Lomonosov Moscow State University, Leninskie Gory, 1-3, Moscow 119992, Russia

^7^ SPE LLC Nanostructured Glass Technology, 101 50 Let Oktjabrja, Saratov 410033, Russia

^8^ Center for Photonics and 2D Materials, Moscow Institute of Physics and Technology, Dolgoprudny 141700, Russia

^9^ Institute of Precision Mechanics and Control of the Russian Academy of Sciences, 24 Rabochaya str., Saratov 410028, Russia

^These authors contributed equally

*The corresponding author, e-mail: d.gorin@skoltech.ru

Table of Contents

[1. Theoretical model 2](#_Toc51253973)

[1.1. Calibration to empty unmodified HC-MOF 2](#_Toc51253974)

[1.2. Empirical extraction of RI for liquid filling 3](#_Toc51253975)

[1.3. Analysis of the coating thickness 4](#_Toc51253976)

[2. Potential setup optimization for measurement of RI at 100 wavelengths 5](#_Toc51253977)

[3. Impact of the liquid sample internal losses on the HC-MOF transmission 6](#_Toc51253978)

[4. Stability of the polymer coating 7](#_Toc51253979)

[5. Transmission characterization of HC-MOFs modified by PEs dissolved in a saline buffer. 11](#_Toc51253980)

[6. Transmission of HC-MOFs filled with a water-BSA solution 12](#_Toc51253981)

[7. Schematic of custom designed liquid cells 12](#_Toc51253982)

[8. Refractive index sensitivity and figure of merit. 13](#_Toc51253983)

[9. Sellmeier fits 16](#_Toc51253984)

[10. Optical dispersion of refractive index for BSA 17](#_Toc51253985)

[11. Refractive index for BSA solutions by the Abbe refractometer and IMOS 17](#_Toc51253986)

[12. Sodium dodecyl sulfate–polyacrylamide gel electrophoresis analysis of BSA samples 20](#_Toc51253987)

[13. Measurement of BSA concentration 21](#_Toc51253988)

[References 22](#_Toc51253989)

## Theoretical model

## Calibration to empty unmodified HC-MOF

The light guidance in HC-MOFs employed in our work has been described in detail in Ref.^1^, and here we briefly describe the theoretical model. The fibre transmission windows are defined by the resonant light interaction with the cladding layers, which can be characterized in terms of the Fabry-Perot model^2,3^. For the sake of simplicity, the HC-MOF cladding is considered as concentric cylindrical layers with air, glass and assembled PAH / PSS bilayers refractive indexes. Within the geometric optics treatment, one can calculate the fibre transmission spectrum as follows^1^

$$T= R^{m}$$

where *R* is the coefficient of reflection from the cladding and *m* is the number of such reflections for the light ray, propagating in the fibre core, on the fibre length *L*. The condition $R=1$ corresponds to the anti-resonance between the Fabry-Perot modes of the cladding and the modes of HC-MOF propagating in the core. Assuming the paraxial approximation, one can derive for the fundamental fibre mode^3^:

$m= \frac{\lambda L}{2d_{0}^{2}n_{0}}$

where *d*_0_ is the core diameter, λ is the wavelength, and *n*_0_ is the refractive index of the fibre core filling (i.e., the air in our case). Next, we follow to P. Yeh^4^ and employ the general transfer-matrix method to calculate *R*, which can be presented as:

$$R {= \left| \frac{M_{21}}{M_{11}} \right|}^{2}$$

Here *M*_11_ and *M*_21_ are the elements of the transfer-matrix that is given by:

$M= \left( \begin{matrix} M_{11} & M_{12} \\ M_{21} & M_{22} \end{matrix} \right)= D_{0}^{-1}\left[ \prod_{l=1}^{N} D_{l}D_{l}D_{l}^{-1} \right]D_{s}$

where *N* is the number of layers.

$$D_{l}= \left\{ \begin{matrix} \left( \begin{matrix} 1 & 1 \\ n_{1}\cos\vartheta_{1} & {-n}_{1}\cos\vartheta_{1} \end{matrix} \right) for TE polarization \\ \left( \begin{matrix} \cos\vartheta_{1} & \cos\vartheta_{1} \\ n_{1} & {-n}_{1} \end{matrix} \right) for TM polarization \end{matrix} \right.$$

$$P_{l}= \left( \begin{matrix} exp(i2\pi n_{1}d_{1}\cos\vartheta_{1}/\lambda) & 0 \\ 0 & exp(-i2\pi n_{1}d_{1}\cos\vartheta_{1}/\lambda) \end{matrix} \right)$$

where *l* stands for the layer index with $l=0$ and $l=s$ corresponding to the fibre core and the external buffer layer, respectively, *d*_1_ and *n*_1_ are the thickness and the refractive index of the *l-th* layer, ϑ_1_ is the angle of light incidence on the interface between *(l*-1*)-th* and *l-th* layers. In the core and all layers filled by air, one can derive cos ϑ_1_ = λ/(2*n*_0_*d*_0_) for the grazing light incidence.

Figure S1 illustrates the dimensions of the unmodified HC-MOF used in the experiment, and Figure S2 shows its transmission spectra. The dispersion of fibre glass RI is adopted from Ref.^1^. The theoretical and experimental transmission spectra are in good agreement that provides us with the reference for further calculations.

**
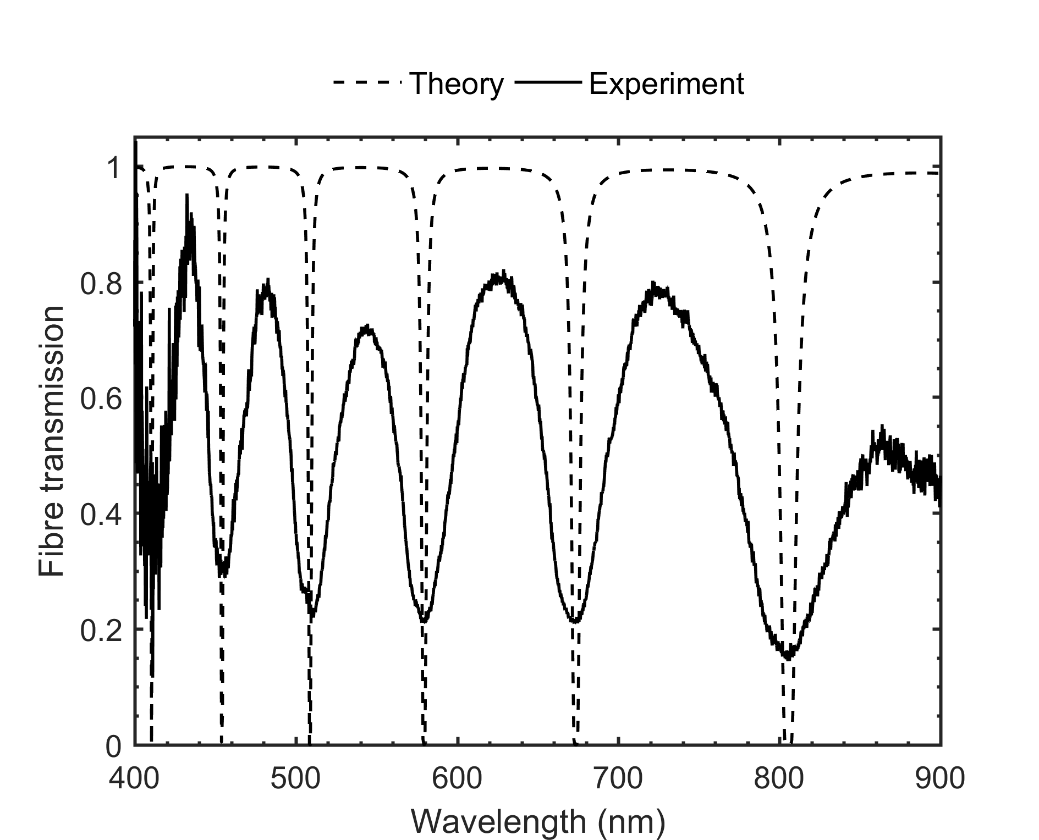

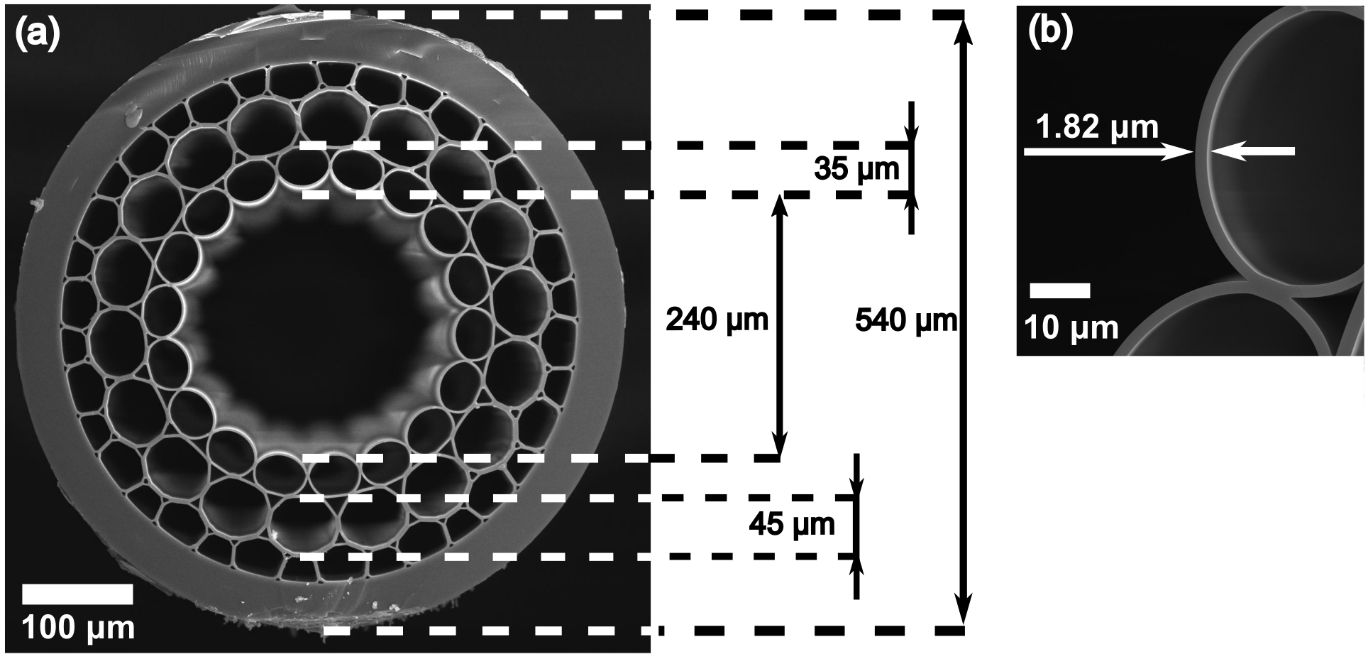
 Figure S1**. SEM images of the unmodified HC-MOF used in the experiments. (a) General view. (b) Inset showing the wall of the central capillary.

**Figure S2.** Theoretical vs experimental transmission for an unmodified and empty HC-MOF with *d*_0_ = 240 μm and *d*_1_ = 1.82 μm.

## Empirical extraction of RI for liquid filling

Figure S3 shows the transmission spectra of the water-filled HC-MOF with different concentration of BSA. Optical dispersion of water is taken as a reference from Ref.^5^ as follows:

$$n_{water}\boldsymbol{=}\sqrt{1+\frac{0.5670093832*\lambda^{2}}{\lambda^{2}-0.004610301794}+\frac{0.1719708856 *\lambda^{2}}{\lambda^{2}-0.01825180155}+\frac{0.01992501582 *\lambda^{2}}{\lambda^{2}-0.02224158904}+\frac{0.1193965424 *\lambda^{2}}{\lambda^{2}-13.27505178}}$$

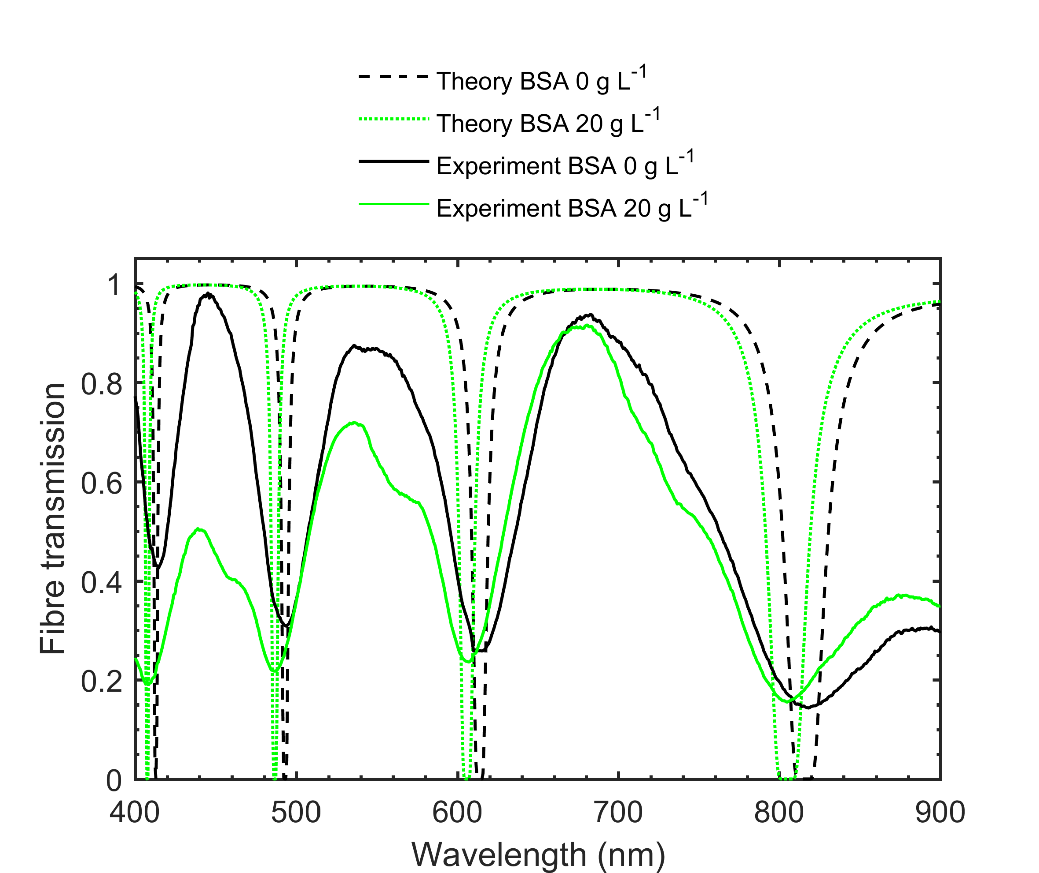
RI of the BSA-water solution can be extracted empirically by adding a correction to $n_{water}$ to obtain coincidence in the positions of transmission minima and maxima (or maxima centroids) for theoretical and experimental transmission spectra (Figure S3).

**Figure S3.** Theoretical and experimental transmission spectra of HC-MOF filled with water and the BSA-water solution.

## Analysis of the coating thickness

The thickness of fibre coating can be evaluated via SEM micrographs of the HC-MOF cross-section (as discussed in the main text) and adjustment of the polymer coating thickness in the theoretical model to get coincidence with the experimental transmission spectrum (Figure S4). Both approaches are in good agreement with each other (Fig. 2a).

**
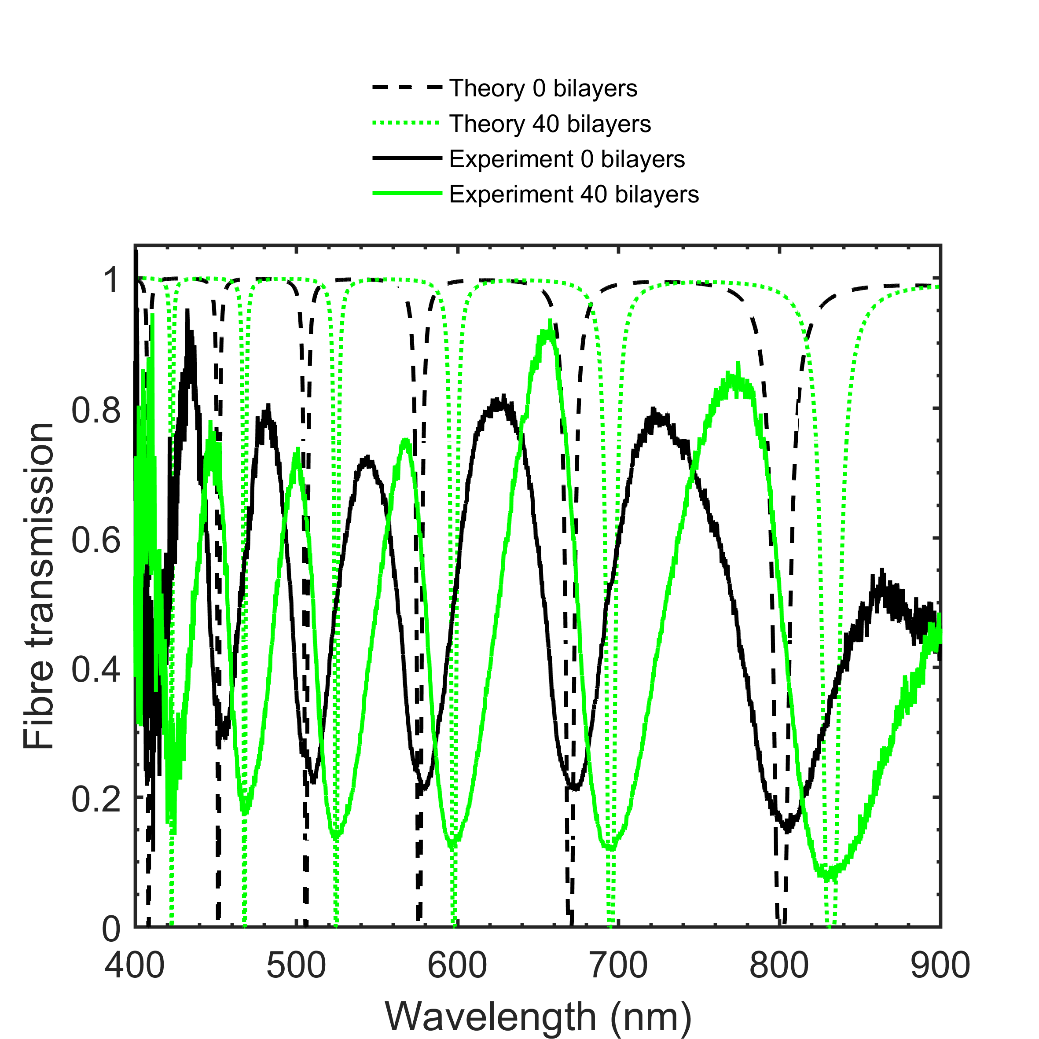
 Figure S4.** Comparison of the experimental fibre transmission spectra with the theoretical ones obtained by adjusting the thickness of the polymer coating (60 nm per 40 polymer bilayers).

## Potential setup optimization for measurement of RI at 100 wavelengths

In practice, the measurement of RI at 100 wavelengths can be realized by the following setup optimization. First, the number of transmission windows in the visible and near-IR spectral domains for a single HC-MOF can be extended up to 10 by increasing the wall thickness of the central capillary and the fibre glass refractive index. Figure S5 illustrates the transmission spectra of a HC-MOF with realistic parameters: the 3-μm capillary wall thickness and the refractive index 1.7. Hence, such HC-MOF makes it possible extraction of RI at 20 spectral points, and 5 functionalized HC-MOF would provide the measurement of RI at 100 wavelengths.

With the current setup, static measurements of RI at 100 wavelengths imply subsequent manual switching of several functionalized HC-MOFs. In order to exclude the probability of failure caused by the manual operation, the setup can be optimized by sealing each fibre in a separate pair of liquid cells then fibre switching in the optical path can be performed automatically via a piezo stage, as shown in Figure S6, or 5 independent optical paths can be created to perform measurements simultaneously with all fibres. These complications, however, do not seem to be expensive or unrealistic because the fabrication of liquid cells with 3D printing is easy and straightforward, and the fibre central capillary size is much larger the optical wavelength that mitigates the problem of optical path alignment. At the same time, its great advantage is automatic and fast measurement of RI optical dispersion in a wide spectral band.

**
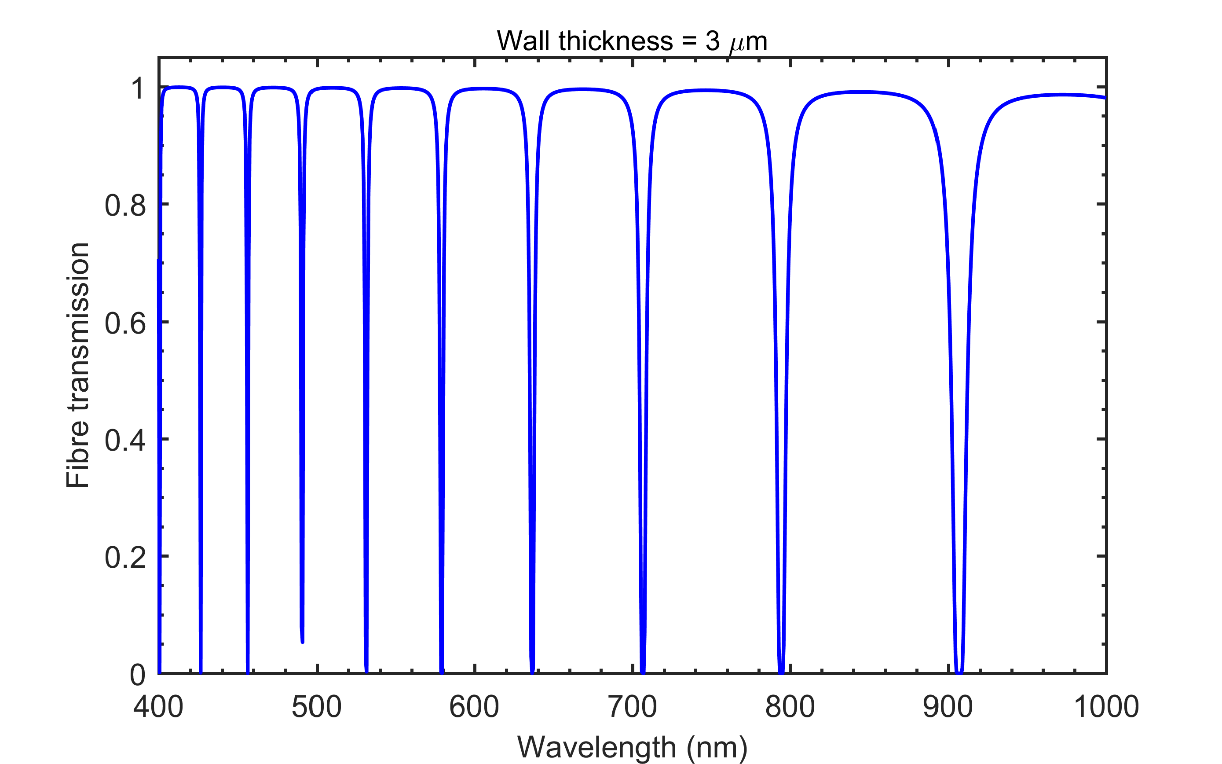
 Figure S5.** Transmission spectra of the water-filled HC-MOF with the 3-μm capillary wall thickness and the refractive index 1.7.


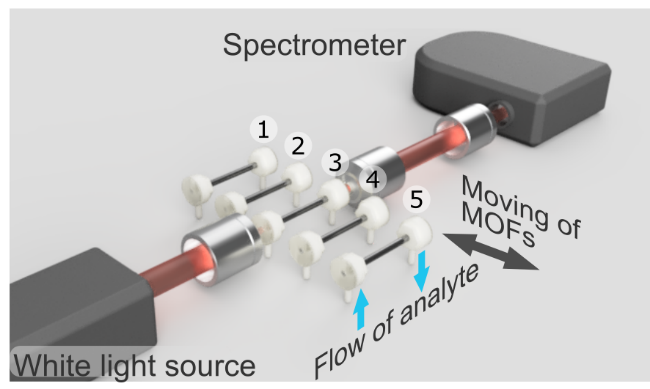


**Figure S6**. Illustration of the automatic fibre switching in the system optical path by a piezo stage.

## Impact of the liquid sample internal losses on the HC-MOF transmission

Increasing optical losses of the sample will lead to eroding the maxima and minima of the fibre transmission spectra, deteriorating the IMOS performance. To provide quantitative evaluations, we plot Fig. S7, showing the fibre transmission spectra of a 6-cm-long fibre for a various imaginary parts of the sample refractive index ($n''$). Importantly, while the maxima are well distinguished up to $n^{''}=0.01$, the minima get markedly eroded for longer wavelengths at $n''\geq0.001$. Thus, IMOS is best performed for sufficiently transparent liquids. We also note that highly absorptive samples are typically sensed with absorption photometry.

**
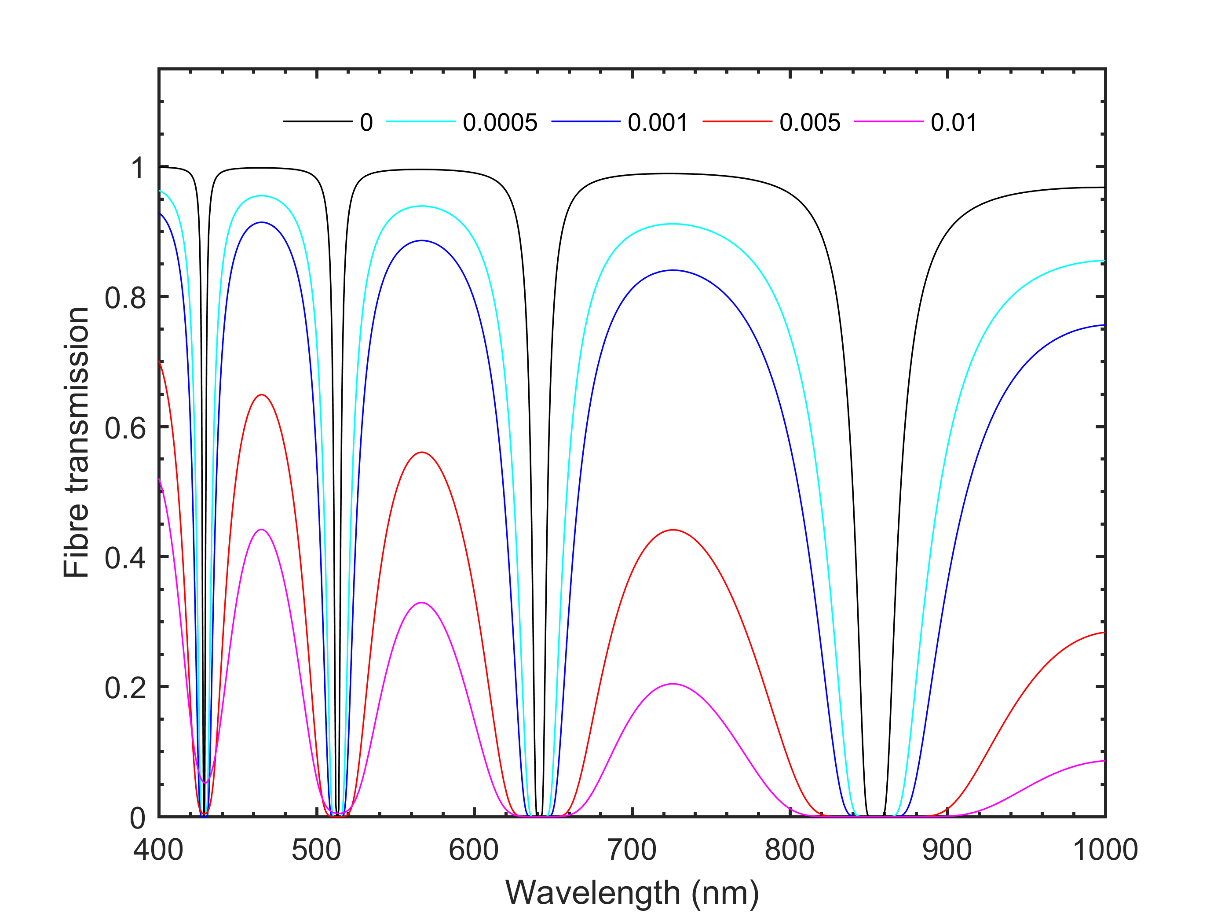
Figure S7.** Fibre transmission spectra for a various imaginary part of the sample refractive index: 0 (black), 0.0005 (cyan), 0.001 (blue), 0.005 (red) and 0.01 (magenta). The real part of RI equals to 1.33.

## Stability of the polymer coating

The stability of the polymer coating has been validated under various conditions. First, we compared the fibre transmission spectra in 1 day and in 4 months after coating (Figure S8). We did not reveal any degradation except for slight variations in the spectral positions of the minima and maxima, which, however, do not exceed 3 nm. We attribute this effect to tiny reduction of the coating thickness caused by drying due to changing ambient humidity. We note that this effect does not have any impact on the IMOS performance since any new measurement requires prior calibration.

Next, we checked the stability of PE layers at various temperatures (from 22 °C to 120 °C), as shown in Figure S9. The value of 37 °C has been chosen as the closest one to the normal body temperature, 60 °C corresponds to an average temperature for protein denaturation^6–9^, 80 °C and 90 °C lye near the glass transition temperature for PAH/PSS layers (similar to rearrangement of PAH/PSS multilayers at capsules)^10–12^ and 120 °C is the temperature that results in evaporation of unbound (free) water^13^. The fibre transmission spectra show that the polymer coating has not been influenced or damaged.

Finally, we verified the stability of PE layers with respect to various pH levels (Figure S10) and ionic strength (Figure S11) of the sample liquid. The measurements show that the polymer coating remains stable since there are no any spectral shifts in fibre transmission in the wide range of pH levels from 4 to 10 as well as concentrations of NaCl up to 1M which is suitable for the majority of bioanalytical applications.

So robust stability with respect to various conditions manifests that any adsorbed molecules can be just washed out at denaturation conditions and the functionalized HC-MOFs can be reused again.

**
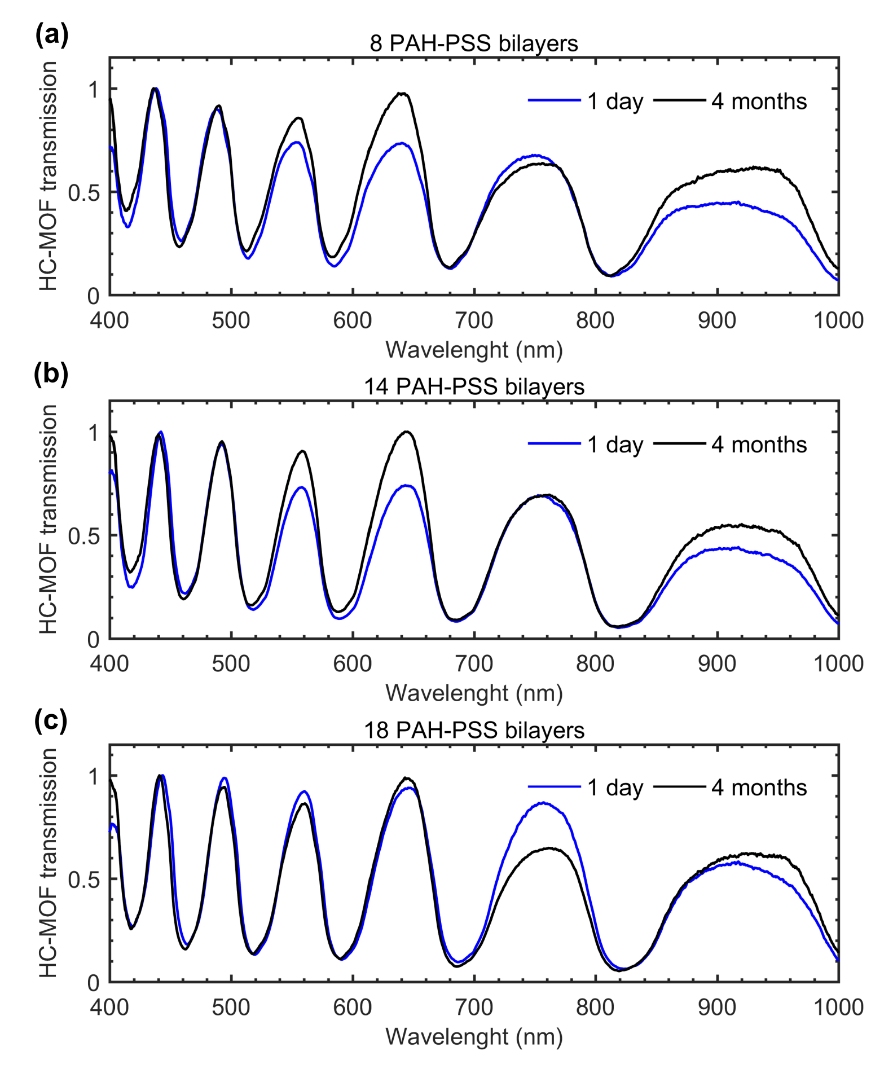
**

**Figure S8**. Transmission spectra of HC-MOFs modified with (a) 8, (b) 14 and (c) 18 PAH/PSS bilayers in 1 day and in 4 months after coating.


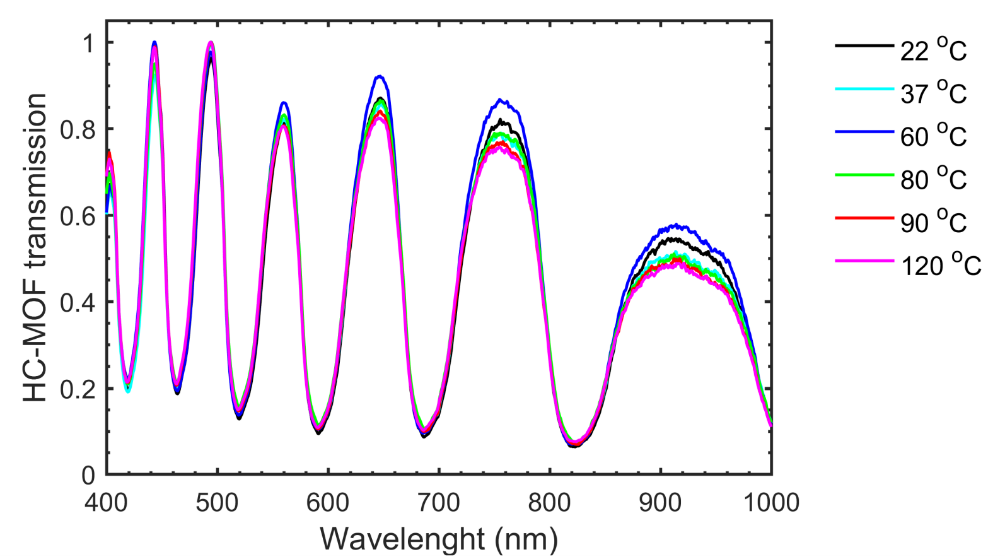


**Figure S9.** Transmission spectra of the HC-MOF modified with 18 PAH/PSS at a various ambient temperature.

**
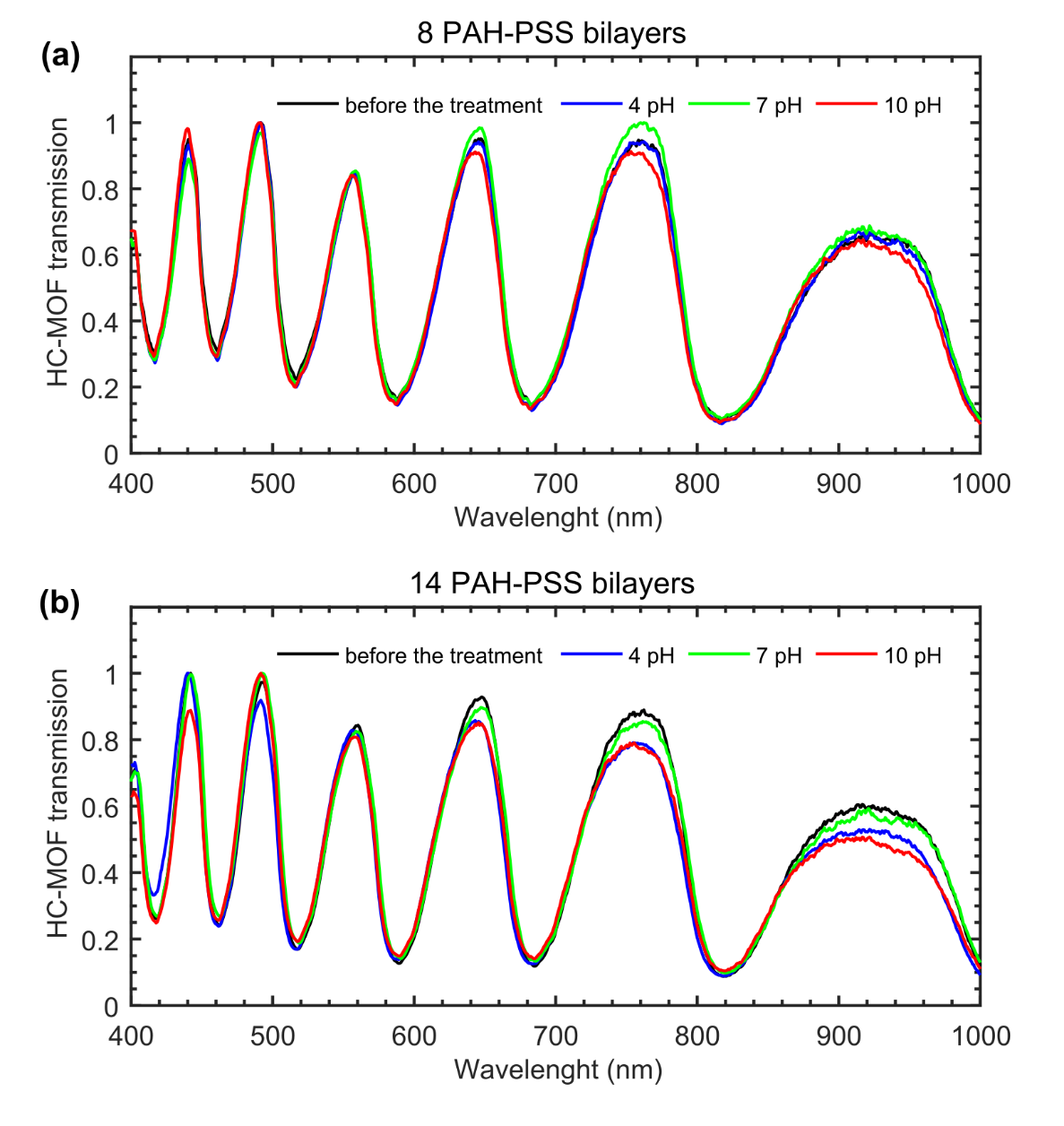
** **Figure S10**. Transmission spectra of the HC-MOFs modified with (a) 8 and (b) 14 PAH/PSS bilayers after streaming liquid with a various pH level during 10 min.

**
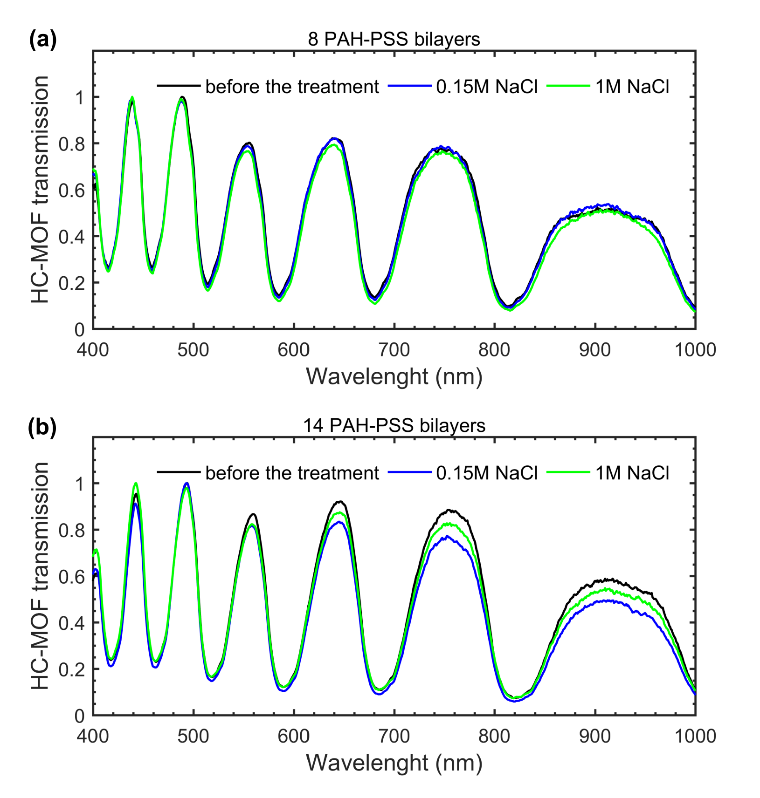
**

**Figure S11**. Impact of ionic strength on the polymer coating. Transmission spectra of the HC-MOFs modified with (a) 8 and (b) 14 PAH/PSS bilayers after streaming liquid with a various concentration of NaCl during 10 min.

## Transmission characterization of HC-MOFs modified by PEs dissolved in a saline buffer.

**
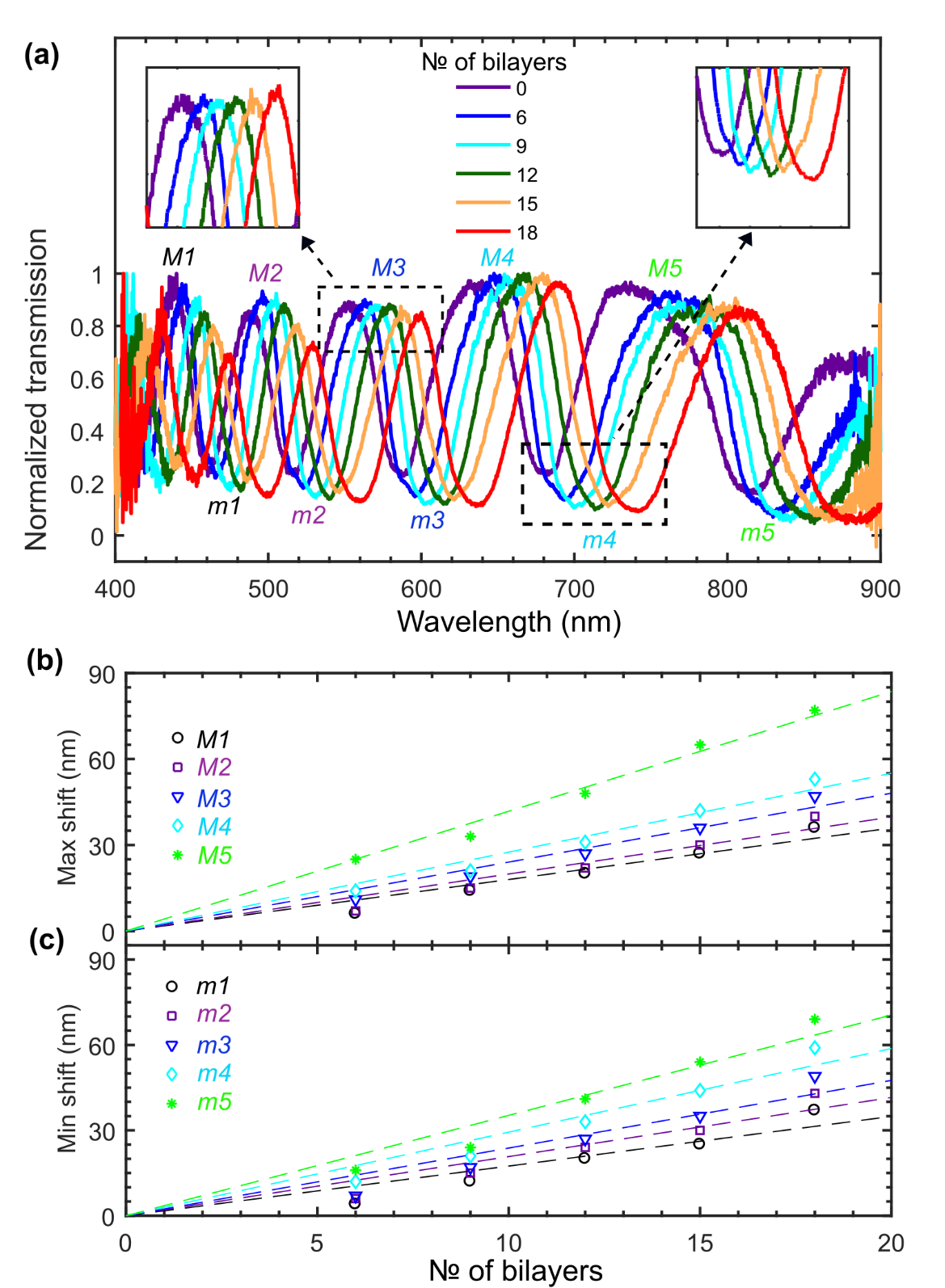
 Figure S12.** Optical characterization of HC-MOFs functionalized by PEs dissolved in saline buffer (0.15 M NaCl). (a) Transmission spectra in the visible range. The results for 6 samples with a different number of PE bilayers are presented. Shifts of maxima (b) and minima (c) of the transmission along with the linear fits.

It is important to note that despite the increased roughness of coating, optical performance and tunability of fibres functionalized by PEs dissolved in a saline buffer remain on a good level (Figure S12). The reason for that is that the roughness scale (Fig. 2) is still much smaller the light wavelength.

## Transmission of HC-MOFs filled with a water-BSA solution

**
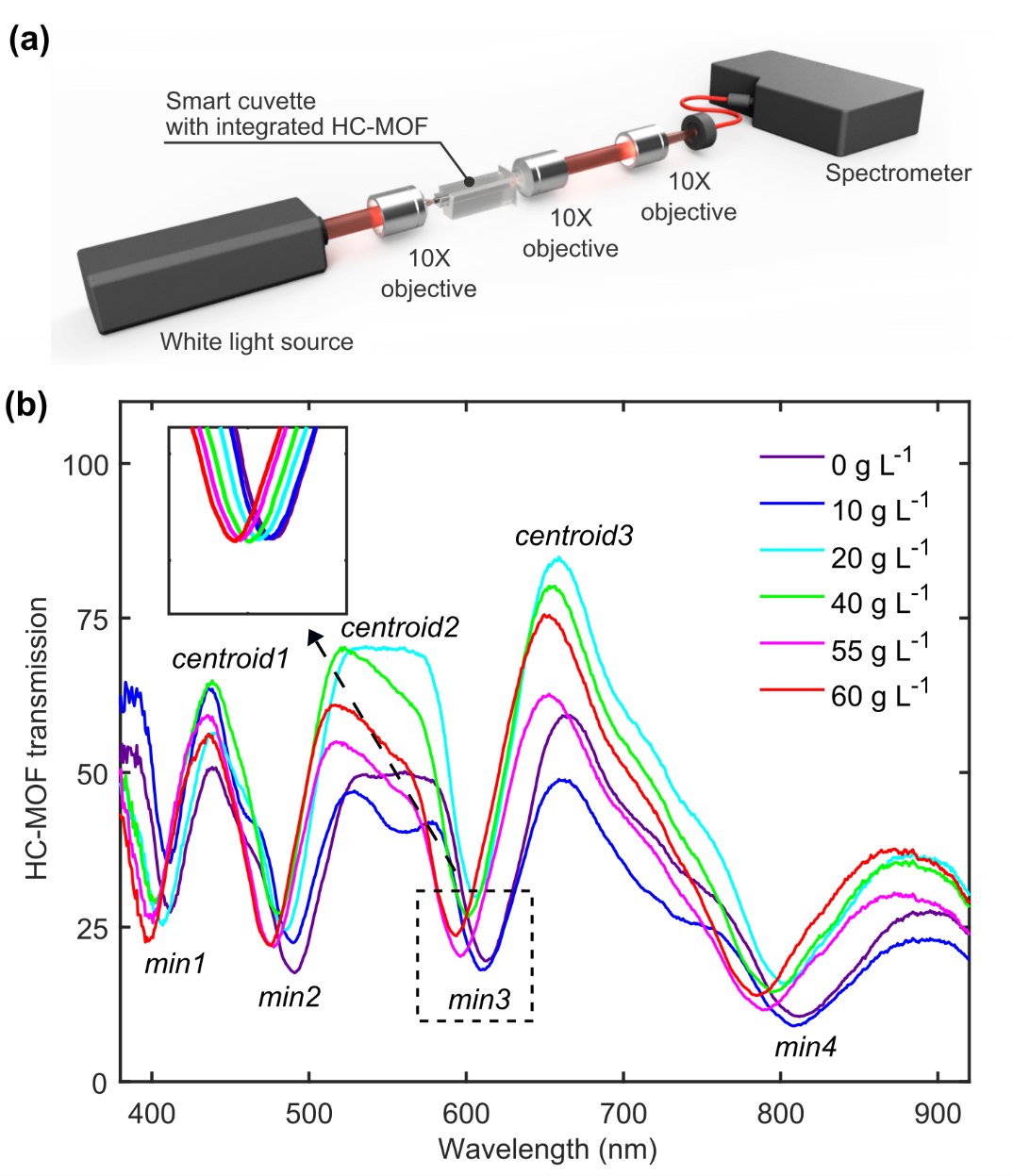
**The set of 6 functionalized HC-MOFs is used to restore the optical dispersion of a water-BSA solution by the combination of the optical setup and the special smart cuvette (see Material and methods and Figure S13a). In addition to fibre holding, the smart cuvette can also control meniscus formation that is important for light coupling when the fibre is filled with liquid. Each of the BSA-filled samples has four well-defined minima of the transmission spectra in the visible spectral range (400 nm – 900 nm) while the peaks are distorted (Figure S13b) and for that reason, we use the centroids of the transmission windows in the analysis.

**Figure S13.** Static measurements of the transmission for HC-MOFs filled with a water-BSA solution. (a) The schematic of the optical setup. (b) The transmission of a non-modified fibre for different concentrations of BSA.

## Schematic of custom designed liquid cells

The detailed schematic of liquid cells is shown in Figure S14. Cells were prepared using the commercially available 3D printer Prusa i3 steel. Its printing tolerance ~0.1 mm totally meets the accuracy requirements for reproducible production of high-quality liquid cells. In total, two liquid cells and HC-MOF sealed in them form a simple and robust structure.

**
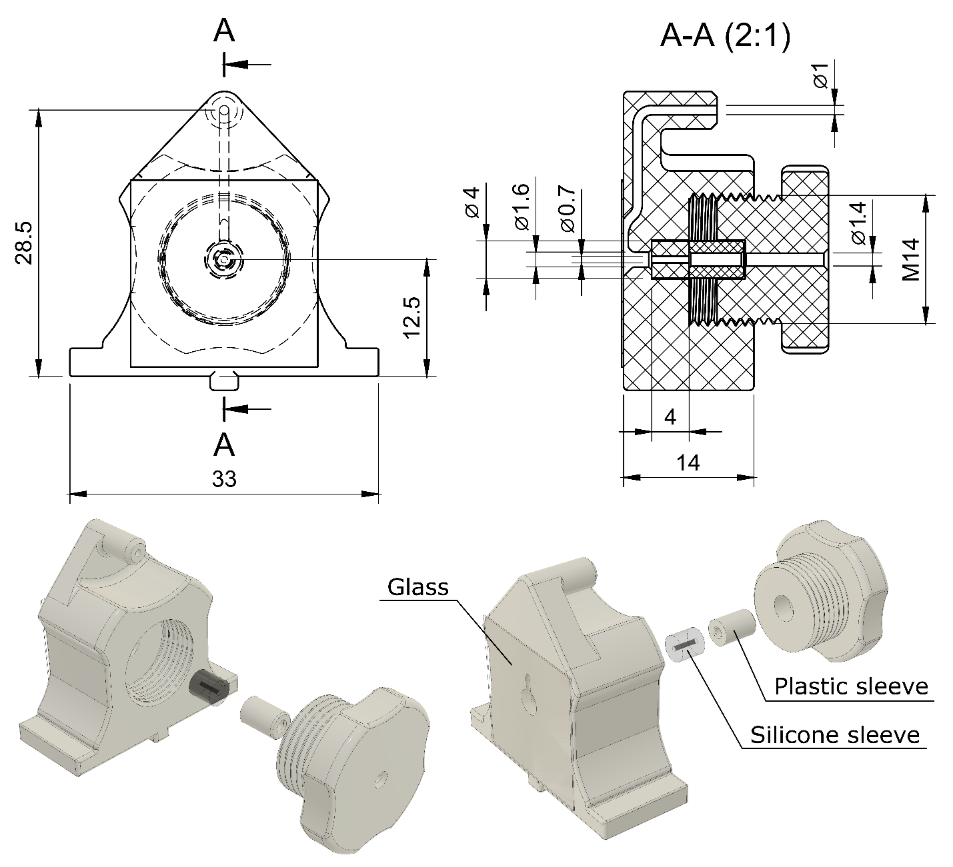
Figure S14.** Schematic of the custom designed liquid cells. Dimensions are denoted in mm.

## Refractive index sensitivity and figure of merit.

The RI sensitivity (RIS) is defined as the ratio of the change in sensor output (e.g., resonant wavelength) to the RI variations. Figure S15 shows the transmission minima as a function of analyte RI along with RIS and FOM. The slope of the linear fit corresponds to RIS as

$$RIS= \frac{\Delta\lambda}{\Delta n}$$

FOM is RIS normalized by the full width at half maximum (FWHM) of a resonance

$$FOM= \frac{RIS}{FWHM}$$

*
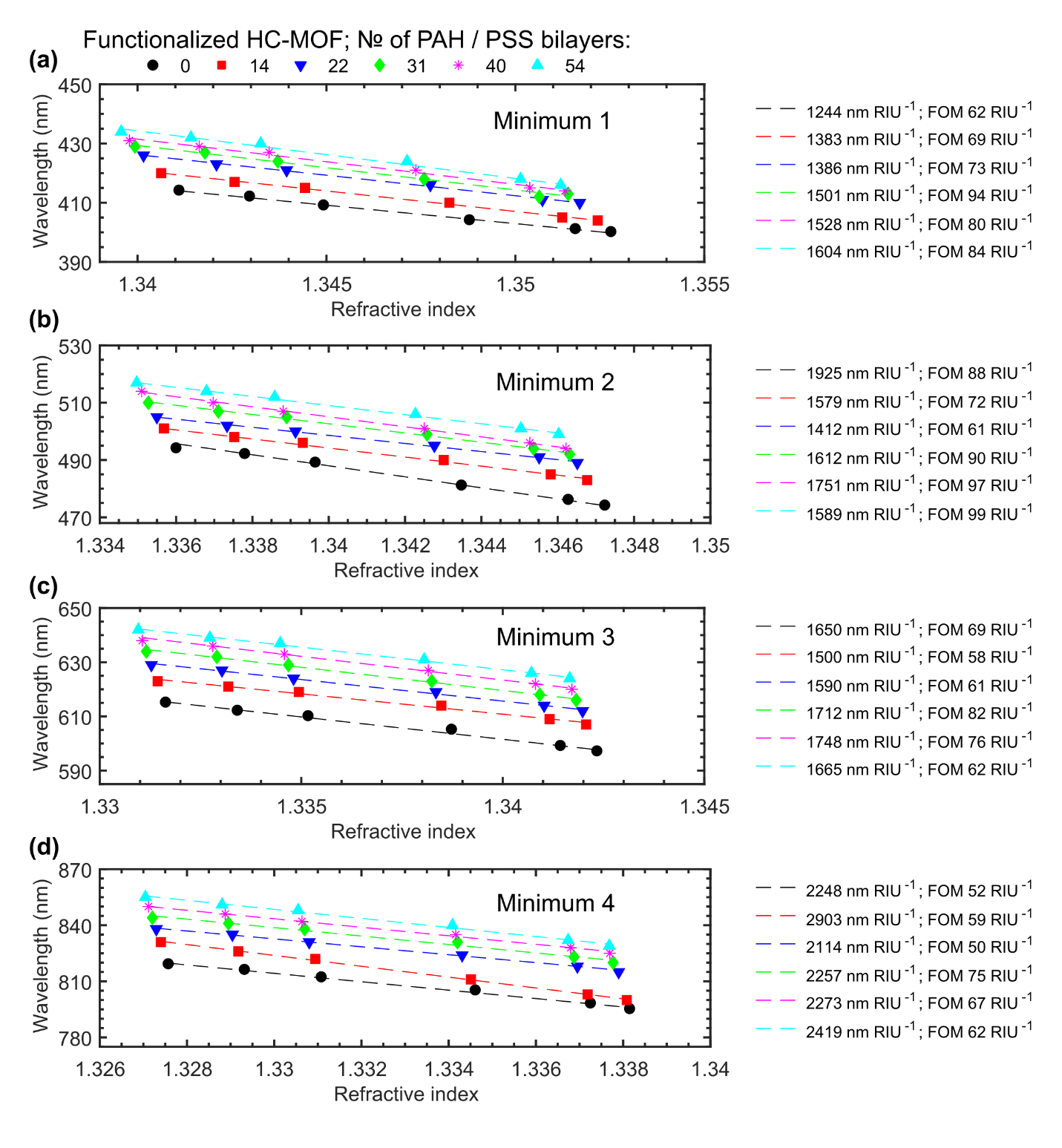
* **Figure S15.** (a,b,c,d) The minima positions extracted from the transmission spectra of HC-MOFs as a function of the filling refractive index obtained by variations in the concentration of BSA. The error bars are smaller than the data points.

To fit the experimental points in Figure S15 we use a linear fit as

$$y=k*x+b$$

Table S1 summarizes the adjusted coefficients.

|  | № of PAH / PSS bilayers | | | | | | | | | | | | | | | | | |
| --- | --- | --- | --- | --- | --- | --- | --- | --- | --- | --- | --- | --- | --- | --- | --- | --- | --- | --- |
| № of Min | 0 | | | 14 | | | 22 | | | 31 | | | 40 | | | 54 | | |
|  | k | b | R^2^ | k | b | R^2^ | k | b | R^2^ | k | b | R^2^ | k | b | R^2^ | k | b | R^2^ |
| Min 1 | -1244 | 2083 | 0.99 | -1383 | 2274 | 0.99 | -1386 | 2283 | 0.99 | -1501 | 2441 | 0.97 | -1528 | 2479 | 0.99 | -1604 | 2584 | 0.99 |
| Min 2 | -1925 | 3067 | 0.99 | -1579 | 2610 | 0.99 | -1412 | 2390 | 0.99 | -1612 | 2663 | 0.99 | -1751 | 2851 | 0.99 | -1589 | 2639 | 0.99 |
| Min 3 | -1650 | 2813 | 0.99 | -1500 | 2621 | 0.99 | -1590 | 2747 | 0.99 | -1712 | 2913 | 0.99 | -1748 | 2966 | 0.99 | -1665 | 2859 | 0.99 |
| Min 4 | -2248 | 3804 | 0.99 | -2903 | 4685 | 0.99 | -2114 | 3644 | 0.99 | -2257 | 3841 | 0.99 | -2273 | 3866 | 0.99 | -2419 | 4066 | 0.99 |

**Table S1**: Coefficients of the linear fit for experimental data in Figure S6. R^2^ is a statistical coefficient showing the fitting performance (R^2^=1 corresponds to perfect fitting).

The standard deviations of the RI are calculated using the spectrometer resolution (1 nm) and the RIS calculated for HC-MOFs as follows

$$\Delta n= \frac{\Delta\lambda}{RIS}=\pm\frac{1}{RIS}$$

|  | № of PAH / PSS bilayers | | | | | |
| --- | --- | --- | --- | --- | --- | --- |
| № of Minimum | 0 | 14 | 22 | 31 | 40 | 54 |
| Minimum 1 | $\pm$0.0008 | $\pm$0.0007 | $\pm$0.0007 | $\pm$0.0007 | $\pm$0.0007 | $\pm$0.0006 |
| Minimum 2 | $\pm$0.0005 | $\pm$0.0006 | $\pm$0.0007 | $\pm$0.0006 | $\pm$0.0006 | $\pm$0.0006 |
| Minimum 3 | $\pm$0.0006 | $\pm$0.0007 | $\pm$0.0006 | $\pm$0.0006 | $\pm$0.0006 | $\pm$0.0006 |
| Minimum 4 | $\pm$0.0004 | $\pm$0.0003 | $\pm$0.0005 | $\pm$0.0004 | $\pm$0.0004 | $\pm$0.0004 |

**Table S2**: Standard deviations of the measured RIs.

## Sellmeier fits

To fit the experimental points for RIs (Figures 5 and 6 in the main text and Figure S18), we use the Sellmeier formula as follows

$$n=\sqrt{1+\frac{A*\lambda^{2}}{\lambda^{2}-B}+\frac{C*\lambda^{2}}{\lambda^{2}-D}+\frac{E*\lambda^{2}}{\lambda^{2}-F}+\frac{G*\lambda^{2}}{\lambda^{2}-H}}$$

where *A, B, C, D, E, F, G, H*  are empirical constants provided in Tables S3 and S4.

| BSA concentration, g L^-1^ | A | B (µm^2^) | C | D (µm^2^) | E | F (µm^2^) | G | H (µm^2^) | R^2^ |
| --- | --- | --- | --- | --- | --- | --- | --- | --- | --- |
| 0 (water) | 32.9 | -38.06 | -55.1 | -186.1 | 23.58 | -29.95 | -0.622 | -9.086 | 0.9981 |
| 10 | -4.752 | -7.967 | 15.35 | 11.42 | -67.2 | -84.06 | 57.37 | 42.26 | 0.9982 |
| 20 | 33.41 | -30.38 | -55.47 | -177.9 | 24.77 | -22.9 | -1.949 | -3.382 | 0.9983 |
| 40 | -3.091 | -1.205 | -64.43 | -62.54 | 63.25 | 66.44 | 5.034 | 7.099 | 0.9985 |
| 55 | 7.53 | 9.666 | -72.92 | -71.31 | 51.52 | 52.97 | 14.64 | 17.78 | 0.9986 |
| 60 | 26.33 | -2.705 | -57.76 | -145.1 | 23.07 | 1.209 | 9.133 | -7.36 | 0.9987 |

**Table S3**: Coefficients of the Selmeier equations used to fit the experimental data in Fig. 5.

| BSA concentration, g L^-1^ | A | B (µm^2^) | C | D (µm^2^) | E | F (µm^2^) | G | H (µm^2^) | R^2^ |
| --- | --- | --- | --- | --- | --- | --- | --- | --- | --- |
| 20 | 19.51 | 11.32 | 67.11 | 17.11 | -19.16 | -34.54 | -66.24 | -109 | 0.9983 |
| 40 | -65.52 | -131.3 | 32.09 | 7.516 | -9.302 | -9.718 | 43.95 | 6.43 | 0.9953 |
| 55 | 38.78 | -64.09 | -49.56 | -246 | 12.99 | -40.79 | -0.996 | -29.93 | 0.9986 |
| 60 | -41.28 | -66.91 | 30.3 | -6.686 | 70.18 | 14.58 | -57.98 | -96.61 | 0.9913 |

**Table S4**: Coefficients of the Selmeier equations used to fit the experimental data in Fig. 6 and Fig. S16, BSA supplied by Sigma-Aldrich.

| BSA concentration, g L^-1^ | A | B (µm^2^) | C | D (µm^2^) | E | F (µm^2^) | G | H (µm^2^) | R^2^ |
| --- | --- | --- | --- | --- | --- | --- | --- | --- | --- |
| 20 | 26.28 | -13.83 | 65.18 | 24.91 | -20.89 | -35.24 | -69.21 | -108.9 | 0.9983 |
| 40 | -68.79 | -123.2 | 30.53 | 12.43 | -5.492 | -17.93 | 45.11 | 11.65 | 0.9958 |
| 55 | 30 | -2.396 | -68.9 | -142.7 | 31.14 | -10.53 | 9.117 | 0.9068 | 0.9973 |

**Table S5**: Coefficients of the Selmeier equations used to fit the experimental data in Fig. S16, BSA supplied by Agat-Med.

##
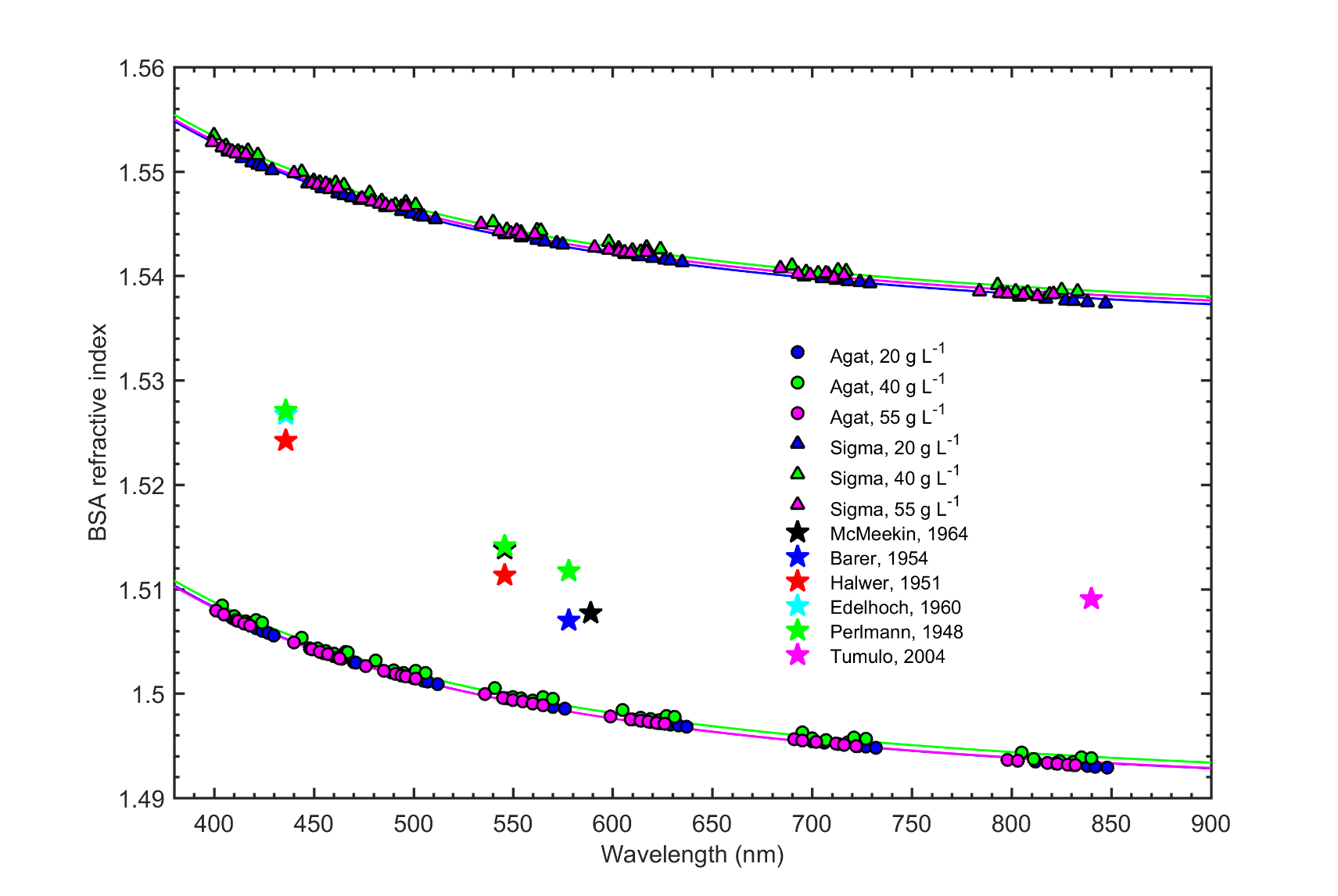
Optical dispersion of refractive index for BSA

**Figure S16.** The refractive index of BSA extracted via the Maxwell Garnett approximation. Circles correspond to the measurements of RI for the BSA supplied by Sigma-Aldrich (>96% purity) and triangles to the BSA supplied by Agat-Med (50% purity), and solid lines mark the Sellmeier fits (Table S4). The extraction is performed for 3 concentrations of BSA to show repeatability of the result. The pentagrams mark BSA RI extracted from Refs^14–19^.

## Refractive index for BSA solutions by the Abbe refractometer and IMOS

| Wavelength, nm | 0 g L^-1^ (water) | Δn | 10 g L^-1^ | Δn | 20 g L^-1^ | Δn |
| --- | --- | --- | --- | --- | --- | --- |
| 480 | 1.3372 | $\pm$0.0001 | 1.3393 | $\pm$0.0001 | 1.3413 | $\pm$0.0007 |
| 486 | 1.3367 | $\pm$0.0003 | 1.3387 | $\pm$0.0002 | 1.3409 | $\pm$0.0001 |
| 546 | 1.334 | $\pm$0.0002 | 1.3361 | $\pm$0.0002 | 1.3382 | $\pm$0.0002 |
| 589 | 1.3324 | $\pm$0.0002 | 1.3346 | $\pm$0.0002 | 1.3366 | $\pm$0.0003 |
| 644 | 1.331 | $\pm$0.0002 | 1.333 | $\pm$0.0001 | 1.3349 | $\pm$0.0003 |
| 656 | 1.3305 | $\pm$0.0002 | 1.3329 | $\pm$0.0001 | 1.3346 | $\pm$0.0001 |
| 680 | 1.3297 | $\pm$0.0002 | 1.3321 | $\pm$0.0003 | 1.334 | $\pm$0.0004 |
| 800 | 1.3279 | $\pm$0.0001 | 1.3297 | $\pm$0.0002 | 1.3318 | $\pm$0.0002 |
| Wavelength, nm | 40 g L^-1^ | Δn | 55 g L^-1^ | Δn | 60 g L^-1^ | Δn |
| 480 | 1.3459 | $\pm$0.0003 | 1.3498 | $\pm$0.0001 | 1.355 | $\pm$0.0002 |
| 486 | 1.3458 | $\pm$0.0001 | 1.3492 | $\pm$0.0002 | 1.3546 | $\pm$0.0001 |
| 546 | 1.3425 | $\pm$0.0002 | 1.3463 | $\pm$0.0001 | 1.3516 | $\pm$0.0001 |
| 589 | 1.3413 | $\pm$0.0001 | 1.3451 | $\pm$0.0001 | 1.3502 | $\pm$0.0002 |
| 644 | 1.3396 | $\pm$0.0001 | 1.3432 | $\pm$0.0001 | 1.3486 | $\pm$0.0002 |
| 656 | 1.3392 | $\pm$0.0002 | 1.3427 | $\pm$0.0002 | 1.3481 | $\pm$0.0002 |
| 680 | 1.3381 | $\pm$0.0002 | 1.3416 | $\pm$0.0003 | 1.347 | $\pm$0.0003 |
| 800 | 1.3362 | $\pm$0.0003 | 1.3398 | $\pm$0.0002 | 1.3451 | $\pm$0.0002 |

**Table S6**: RI measured by the Abbe refractometer at 24°C. Δn corresponds to the standard deviation.

| λ, nm | 10  g L^-1^ | Δn | λ, nm | 20  g L^-1^ | Δn | λ, nm | 40  g L^-1^ | Δn |
| --- | --- | --- | --- | --- | --- | --- | --- | --- |
| 412 | 1.3430 | $\pm$0.0008 | 409 | 1.3449 | $\pm$0.0008 | 404 | 1.3488 | $\pm$0.0008 |
| 417 | 1.3426 | $\pm$0.0007 | 415 | 1.3444 | $\pm$0.0007 | 410 | 1.3483 | $\pm$0.0007 |
| 423 | 1.3421 | $\pm$0.0007 | 421 | 1.3439 | $\pm$0.0007 | 416 | 1.3477 | $\pm$0.0007 |
| 427 | 1.3418 | $\pm$0.0007 | 424 | 1.3437 | $\pm$0.0007 | 418 | 1.3476 | $\pm$0.0007 |
| 429 | 1.3416 | $\pm$0.0007 | 427 | 1.3435 | $\pm$0.0007 | 421 | 1.3474 | $\pm$0.0007 |
| 432 | 1.3414 | $\pm$0.0006 | 430 | 1.3433 | $\pm$0.0006 | 424 | 1.3471 | $\pm$0.0006 |
| 451 | 1.3401 | - | 448 | 1.3420 | - | 444 | 1.3457 | - |
| 457 | 1.3397 | - | 455 | 1.3416 | - | 452 | 1.3452 | - |
| 462 | 1.3394 | - | 460 | 1.3413 | - | 456 | 1.3449 | - |
| 466 | 1.3392 | - | 464 | 1.3410 | - | 460 | 1.3447 | - |
| 471 | 1.3389 | - | 470 | 1.3407 | - | 466 | 1.3443 | - |
| 473 | 1.3388 | - | 471 | 1.3406 | - | 467 | 1.3443 | - |
| 492 | 1.3378 | $\pm$0.0008 | 489 | 1.3397 | $\pm$0.0008 | 481 | 1.3435 | $\pm$0.0008 |
| 498 | 1.3375 | $\pm$0.0007 | 496 | 1.3393 | $\pm$0.0007 | 490 | 1.3430 | $\pm$0.0007 |
| 502 | 1.3373 | $\pm$0.0007 | 500 | 1.3391 | $\pm$0.0007 | 495 | 1.3428 | $\pm$0.0007 |
| 507 | 1.3371 | $\pm$0.0007 | 505 | 1.3389 | $\pm$0.0007 | 499 | 1.3426 | $\pm$0.0007 |
| 510 | 1.3370 | $\pm$0.0007 | 507 | 1.3388 | $\pm$0.0007 | 501 | 1.3425 | $\pm$0.0007 |
| 514 | 1.3368 | $\pm$0.0006 | 512 | 1.3386 | $\pm$0.0006 | 506 | 1.3423 | $\pm$0.0006 |
| 550 | 1.3354 | - | 547 | 1.3372 | - | 541 | 1.3408 | - |
| 557 | 1.3351 | - | 555 | 1.3369 | - | 550 | 1.3405 | - |
| 562 | 1.3350 | - | 560 | 1.3367 | - | 554 | 1.3403 | - |
| 567 | 1.3348 | - | 565 | 1.3366 | - | 560 | 1.3401 | - |
| 572 | 1.3346 | - | 570 | 1.3364 | - | 565 | 1.3400 | - |
| 578 | 1.3344 | - | 576 | 1.3362 | - | 570 | 1.3398 | - |
| 612 | 1.3334 | $\pm$0.0008 | 610 | 1.3352 | $\pm$0.0008 | 605 | 1.3387 | $\pm$0.0008 |
| 621 | 1.3332 | $\pm$0.0007 | 619 | 1.3349 | $\pm$0.0007 | 614 | 1.3385 | $\pm$0.0007 |
| 627 | 1.3330 | $\pm$0.0007 | 624 | 1.3348 | $\pm$0.0007 | 619 | 1.3383 | $\pm$0.0007 |
| 632 | 1.3329 | $\pm$0.0007 | 629 | 1.3347 | $\pm$0.0007 | 623 | 1.3382 | $\pm$0.0007 |
| 636 | 1.3328 | $\pm$0.0007 | 633 | 1.3346 | $\pm$0.0007 | 627 | 1.3382 | $\pm$0.0007 |
| 639 | 1.3327 | $\pm$0.0006 | 637 | 1.3345 | $\pm$0.0006 | 631 | 1.3380 | $\pm$0.0006 |
| 703 | 1.3313 | - | 700 | 1.3331 | - | 695 | 1.3366 | - |
| 708 | 1.3312 | - | 706 | 1.3330 | - | 700 | 1.3365 | - |
| 716 | 1.3311 | - | 713 | 1.3328 | - | 707 | 1.3363 | - |
| 725 | 1.3309 | - | 723 | 1.3326 | - | 718 | 1.3361 | - |
| 729 | 1.3308 | - | 727 | 1.3325 | - | 721 | 1.3361 | - |
| 735 | 1.3307 | - | 732 | 1.3325 | - | 727 | 1.3360 | - |
| 816 | 1.3293 | $\pm$0.0008 | 812 | 1.3311 | $\pm$0.0008 | 805 | 1.3346 | $\pm$0.0008 |
| 826 | 1.3292 | $\pm$0.0007 | 822 | 1.3309 | $\pm$0.0007 | 811 | 1.3345 | $\pm$0.0007 |
| 835 | 1.3290 | $\pm$0.0007 | 831 | 1.3308 | $\pm$0.0007 | 824 | 1.3343 | $\pm$0.0007 |
| 841 | 1.3290 | $\pm$0.0007 | 838 | 1.3307 | $\pm$0.0007 | 831 | 1.3342 | $\pm$0.0007 |
| 846 | 1.3289 | $\pm$0.0007 | 842 | 1.3306 | $\pm$0.0007 | 835 | 1.3342 | $\pm$0.0007 |
| 851 | 1.3288 | $\pm$0.0006 | 848 | 1.3305 | $\pm$0.0006 | 840 | 1.3341 | $\pm$0.0006 |

| λ, nm | 55  g L^-1^ | Δn | λ, nm | 60  g L^-1^ | Δn |
| --- | --- | --- | --- | --- | --- |
| 401 | 1.3516 | $\pm$0.0008 | 400 | 1.3525 | $\pm$0.0008 |
| 405 | 1.3512 | $\pm$0.0007 | 404 | 1.3522 | $\pm$0.0007 |
| 411 | 1.3507 | $\pm$0.0007 | 410 | 1.3517 | $\pm$0.0007 |
| 412 | 1.3506 | $\pm$0.0007 | 413 | 1.3514 | $\pm$0.0007 |
| 415 | 1.3504 | $\pm$0.0007 | 414 | 1.3513 | $\pm$0.0007 |
| 418 | 1.3501 | $\pm$0.0006 | 416 | 1.3512 | $\pm$0.0006 |
| 440 | 1.3485 | - | 438 | 1.3495 | - |
| 449 | 1.3479 | - | 448 | 1.3488 | - |
| 453 | 1.3476 | - | 451 | 1.3487 | - |
| 457 | 1.3474 | - | 455 | 1.3484 | - |
| 462 | 1.3471 | - | 460 | 1.3481 | - |
| 463 | 1.3470 | - | 462 | 1.3479 | - |
| 476 | 1.3463 | $\pm$0.0008 | 474 | 1.3473 | $\pm$0.0008 |
| 485 | 1.3458 | $\pm$0.0007 | 483 | 1.3468 | $\pm$0.0007 |
| 491 | 1.3455 | $\pm$0.0007 | 489 | 1.3465 | $\pm$0.0007 |
| 494 | 1.3454 | $\pm$0.0007 | 492 | 1.3463 | $\pm$0.0007 |
| 496 | 1.3453 | $\pm$0.0007 | 494 | 1.3462 | $\pm$0.0007 |
| 501 | 1.3450 | $\pm$0.0006 | 499 | 1.3460 | $\pm$0.0006 |
| 536 | 1.3435 | - | 535 | 1.3445 | - |
| 545 | 1.3432 | - | 544 | 1.3441 | - |
| 550 | 1.3430 | - | 549 | 1.3440 | - |
| 555 | 1.3428 | - | 553 | 1.3438 | - |
| 560 | 1.3427 | - | 559 | 1.3436 | - |
| 565 | 1.3425 | - | 563 | 1.3434 | - |
| 599 | 1.3414 | $\pm$0.0008 | 597 | 1.3424 | $\pm$0.0008 |
| 609 | 1.3412 | $\pm$0.0007 | 607 | 1.3421 | $\pm$0.0007 |
| 614 | 1.3410 | $\pm$0.0007 | 612 | 1.3420 | $\pm$0.0007 |
| 618 | 1.3409 | $\pm$0.0007 | 616 | 1.3418 | $\pm$0.0007 |
| 622 | 1.3408 | $\pm$0.0007 | 620 | 1.3417 | $\pm$0.0007 |
| 626 | 1.3407 | $\pm$0.0006 | 624 | 1.3417 | $\pm$0.0006 |
| 691 | 1.3392 | - | 690 | 1.3401 | - |
| 695 | 1.3391 | - | 693 | 1.3400 | - |
| 702 | 1.3390 | - | 700 | 1.3399 | - |
| 712 | 1.3388 | - | 711 | 1.3397 | - |
| 716 | 1.3387 | - | 715 | 1.3396 | - |
| 722 | 1.3386 | - | 720 | 1.3395 | - |
| 798 | 1.3373 | $\pm$0.0008 | 795 | 1.3382 | $\pm$0.0008 |
| 803 | 1.3372 | $\pm$0.0007 | 800 | 1.3381 | $\pm$0.0007 |
| 818 | 1.3370 | $\pm$0.0007 | 815 | 1.3379 | $\pm$0.0007 |
| 823 | 1.3369 | $\pm$0.0007 | 820 | 1.3378 | $\pm$0.0007 |
| 828 | 1.3368 | $\pm$0.0007 | 825 | 1.3377 | $\pm$0.0007 |
| 832 | 1.3367 | $\pm$0.0006 | 829 | 1.3377 | $\pm$0.0006 |

**Table S7**: RIs provided by IMOS (Table S3). We evaluated error bars for transmission minima only.

## Sodium dodecyl sulfate–polyacrylamide gel electrophoresis analysis of BSA samples

BSA samples (1 µg) were separated by sodium dodecyl sulfate -polyacrylamide gel electrophoresis (SDS-PAGE) according to their molecular weight. Albumin standards (Thermo Scientific, 23208) were used as controls in the amount of 1, 1.5 and 2 µg. Proteins were mixed with 6 x Laemmli Loading buffer (6% sodium dodecyl sulfate (SDS), 9% 2-mercaptoethanol, 4.8% glycerol, 0.03% bromophenol blue, 0.375 M Tris-HCl (pH = 6.8), denatured at 95 °C for 10 min and loaded on a gel. SDS- polyacrylamide gel consisted of two different gels: stacking gel (2 ml mQ water, 1 ml Acrylamide / Bis-acrylamide (30%, 29:1), 325 µL Tris-HCl (1 M, pH 6.8), 30 µL 10% SDS, 30 µL 10% Ammonium persulfate (APS), 5 µL TEMED (N, N, N′, N′-tetramethylethylenediamine) was used for concentrating proteins after loading, and separating gel (2 ml mQ water, 1.7 ml Acrylamide / Bis-acrylamide (30%, 29:1), 325 µL Tris-HCl (1.5 M, pH 8.8), 50 µL 10% SDS, 50 µL 10% Ammonium persulfate (APS), 5 µL TEMED (N, N, N′, N′-tetramethyl ethylene-diamine), which allowed separating proteins according their MW. The running buffer was SDS-Tris-Glycine. The running voltage was 80 V for 5 min, then 135 V for 40 min. Protein bands in the gel were visualized by staining with Coomassie brilliant blue R250 (Figure S17).


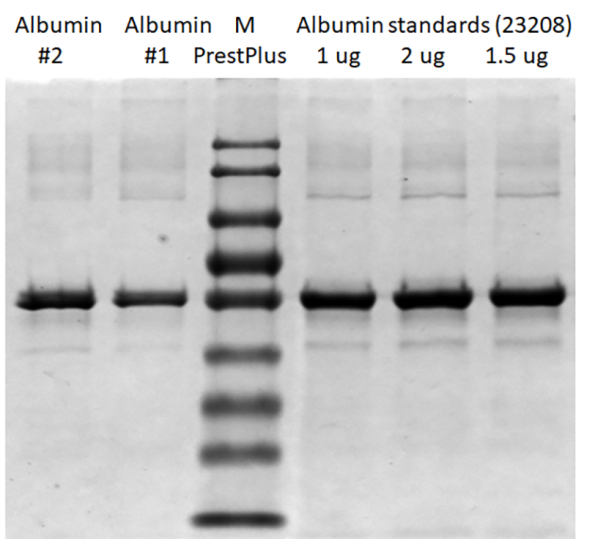


**Figure S17**. SDS-PAGE gel electrophoresis of Albumin samples. M – Prestained Plus protein ladder (MW 250, 130, 100, 70, 55, 35, 25, 15, 10 kDa). BSA standards were from the Thermo Scientific kit (23208).

## Measurement of BSA concentration

BSA samples (20±0.1 mg) were dissolved in RIPA (Radioimmunoprecipitation) buffer (Thermo Scientific, 8900) to the final concentration of 1 mg mL^-1^. To prepare BSA standards we performed serial dilutions of Albumin Standard (BSA) ampules (Thermo Scientific, 23208) using RIPA buffer to the final BSA concentration (mg mL^-1^): 1.5; 1; 0.75; 0.5; 0.25; 0.125; 0.025; 0 mg mL^-1^. To measure BSA concentration we used BCA (Bicinchoninic acid) assay. Working Reagent (WR) was prepared by mixing 50 parts of BCA Reagent A with 1 part of BCA Reagent B (Thermo Scientific, 23225). After that, we mixed 25 µL of BSA sample solution and 200 µL of the WR in 96 well plates using a shaker for 30 sec. Then the covered plate was incubated at 37 °C for 30 minutes and the absorbance was measured at 562 nm using NanoDrop™ One Microvolume UV-Vis Spectrophotometer. Based on the measurements (Table S8), we made a calibration curve (Figure S18) and calculated the BSA amount in samples.

| BSA standard,  concentration mg mL^-1^ | Absorbance at 562 nm | | |
| --- | --- | --- | --- |
| 0 | 0.01 | 0.02 | 0.01 |
| 0.025 | 0.12 | 0.2 | 0.1 |
| 0.125 | 0.44 | 0.5 | 0.41 |
| 0.25 | 0.82 | 0.78 | 0.84 |
| 0.5 | 1.51 | 1.6 | 1.55 |
| 0.75 | 1.96 | 1.89 | 1.95 |
| 1 | 2.67 | 2.59 | 2.62 |
| 1.5 | 3.43 | 3.45 | 3.42 |
| Studied samples | | | |
|  | | | |
| #1(Agat) – 0.49±0.06 | 1.619 | 1.55 | 1.50 |
| #2 (Sigma) – 1.15±0.11 | 2.828 | 2.76 | 2.9 |

**Table S8.** The absorbance of BSA samples in BSA assay at 562 nm.


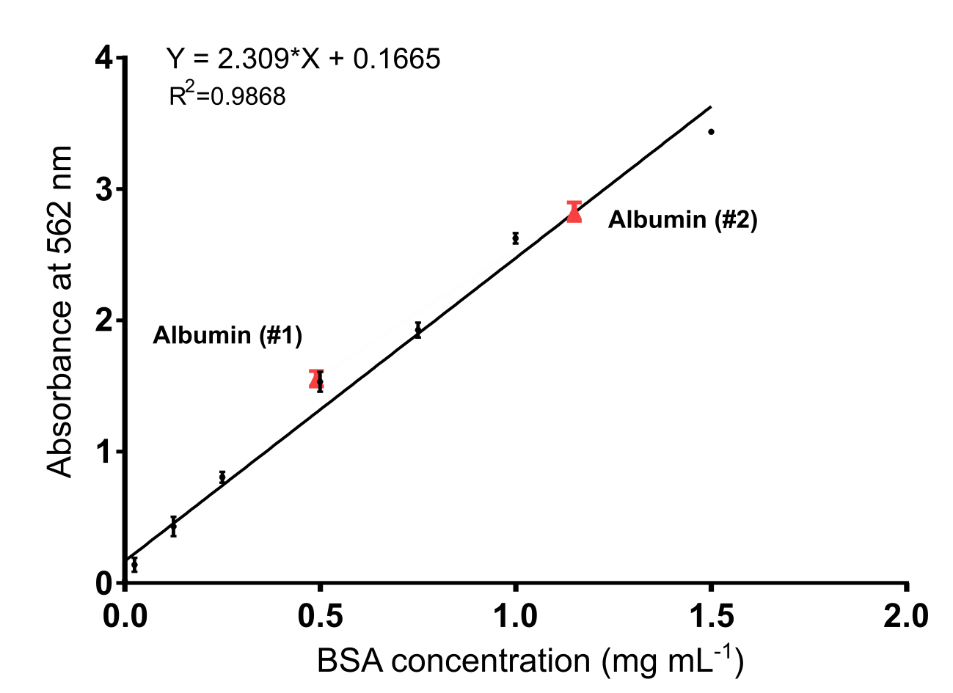


**Figure S18.** The standard curve of BSA measured at 562 nm. The red square marked the experimental samples.

SDS-PAGE analysis demonstrated that both BSA samples contain a protein that has the same mobility and comparable protein purity as the BSA standard (Figure S18). However, measurement of the protein concentration using BCA assay showed that in sample #1 BSA content is only 0.49±0.06, while sample #2 was consistent with the declared BSA content (Table S8).

## References

1. Noskov, R. E. *et al.* Enabling magnetic resonance imaging of hollow-core microstructured optical fibers via nanocomposite coating. *Optics Express* **27**, 9868–9878 (2019).

2. Litchinitser, N. M., Abeeluck, A. K., Headley, C. & Eggleton, B. J. Antiresonant reflecting photonic crystal optical waveguides. *Optics Letters* **27**, 1592–1594 (2002).

3. Zheltikov, A. M. METHODOLOGICAL NOTES: Colors of thin films, antiresonant phenomena in optical systems, and the limiting loss of modes in hollow optical waveguides. *Physics-Uspekhi* **51**, 591–600 (2008).

4. Yeh, P. Optical waves in layered media: Ch 11 b. in *Optical waves in layered media* (2005).

5. Daimon, M. & Masumura, A. Measurement of the refractive index of distilled water from the near-infrared region to the ultraviolet region. *Applied Optics* **46**, 3811–3820 (2007).

6. Rochu, D., Ducret, G., Ribes, F., Vanin, S. & Masson, P. Capillary zone electrophoresis with optimized temperature control for studying thermal denaturation of proteins at various pH. *Electrophoresis* **20**, 1586–1594 (1999).

7. Poklar, N. & Vesnaver, G. Thermal Denaturation of Proteins Studied by UV Spectroscopy. *Journal of Chemical Education* **77**, 380 (2000).

8. Schön, A., Clarkson, B. R., Jaime, M. & Freire, E. Temperature stability of proteins: Analysis of irreversible denaturation using isothermal calorimetry. *Proteins* **85**, 2009–2016 (2017).

9. Stabursvik, E. & Martens, H. Thermal denaturation of proteins in Post rigor muscle tissue as studied by differential scanning calorimetry. *Journal of the Science of Food and Agriculture* **31**, 1034–1042 (1980).

10. Hodge, I. ~M. Enthalpy relaxation and recovery in amorphous materials. *Journal of Non Crystalline Solids* **169**, 211–266 (1994).

11. Hutchinson, J. M. Studying the Glass Transition by DSC and TMDSC. *Journal of Thermal Analysis and Calorimetry* **72**, 619–629 (2003).

12. Glinel, K. *et al.* Responsive polyelectrolyte multilayers. *Colloids and Surfaces A: Physicochemical and Engineering Aspects* **303**, 3–13 (2007).

13. Voskanyan, A. A., Ho, C.-K. & Chan, K. Y. 3D δ-MnO2 nanostructure with ultralarge mesopores as high-performance lithium-ion battery anode fabricated via colloidal solution combustion synthesis. *Journal of Power Sources* **421**, 162–168 (2019).

14. Barer, R. & Joseph, S. Refractometry of Living Cells: Part 1 Basic Principles. *Quarterly Journal of Microscopial Science1* **95**, 399–423 (1954).

15. Halwer, M., Nutting, G. C. & Brice, B. A. Molecular Weight of Lactoglobulin, Ovalbumin, Lysozyme and Serum Albumin by Light Scattering. *Journal of the American Chemical Society* **73**, 2786–2790 (1951).

16. Edelhoch, H. The properties of thyroglobulin. I. The effects of alkali. *The Journal of biological chemistry* **235**, 1326–1334 (1960).

17. Perlmann, G. E. & Longsworth, L. G. The Specific Refractive Increment of Some Purified Proteins. *Journal of the American Chemical Society* **70**, 2719–2724 (1948).

18. Tumolo, T., Angnes, L. & Baptista, M. S. Determination of the refractive index increment (*dn/dc*) of molecule and macromolecule solutions by surface plasmon resonance. *Analytical Biochemistry* **333**, 273–279 (2004).

19. McMeekin, T. L., Groves, M. L. & Hipp, N. J. Refractive Indices of Amino Acids, Proteins, and Related Substances. in *Amino Acids and Serum Proteins* (ed Stekol, J.A.) (Washington: American Chemical Society, 1964). doi:10.1021/ba-1964-0044.ch004
